# Uptake and Biotransformation Govern the Toxicity of Reactive Acrylamides in an *In Vivo* Zebrafish Embryo Model: Implications for NAM-Based Hazard Assessment

**DOI:** 10.1021/acs.est.5c10178

**Published:** 2026-02-18

**Authors:** Nico Grasse, Stefan Scholz, Thorsten Reemtsma, Qiuguo Fu

**Affiliations:** † Department of Environmental Analytical Chemistry, Helmholtz-Centre for Environmental Research − UFZ, Permoserstraße 15, 04318 Leipzig, Germany; ‡ Department of Ecotoxicology, Helmholtz-Centre for Environmental Research − UFZ, Permoserstraße 15, 04318 Leipzig, Germany; § Institute for Analytical Chemistry, University of Leipzig, Linnéstraße 3, 04103 Leipzig, Germany

**Keywords:** reactive toxicity, electrophiles, biotransformation, new approach methodologies, water, bioaccumulation, oxidative stress

## Abstract

Acrylamides are widely used in polymer manufacturing and adhesives, and their electrophilic nature raises toxicological concerns when released into aquatic environments. However, their physicochemical diversity complicates the prediction of environmental fate and toxicity. To elucidate the main drivers of their toxicity in aquatic organisms, we investigated ten structurally diverse monomeric acrylamides and methacrylamides in zebrafish embryos (*Danio rerio*) (ZFE). Acute toxicity varied over 2 orders of magnitude (0.16–33 mM) and showed a moderate correlation with hydrophobicity (log*K*_lipw_, *R* ^2^ = 0.87) and intrinsic electrophilic reactivity (log*k*_GSH_). Measured bioconcentration factors of highly polar, reactive compounds (e.g., NMBA) for ZFE deviated up to 16-fold from model predictions, indicating limited uptake or significant biotransformation. This indicates an impact of toxicokinetics on their *in vivo* toxicity. UPLC-HRMS-based nontarget screening revealed 90 transformation products across the ten compounds. Glutathione conjugation and mercapturic acid formation were dominant pathways, with mercapturic acid-taurine conjugates observed for eight compounds, suggesting a previously undescribed detoxification mechanism for (meth)­acrylamides in the ZFE. Our results highlight the need to integrate toxicokinetic data into hazard assessment of electrophilic compounds, as *in vitro* assays may overestimate risks. The ZFE provides a mechanistically informative *in vivo* model to reduce misclassifications, especially for polar and reactive chemicals.

## Graphical abstract

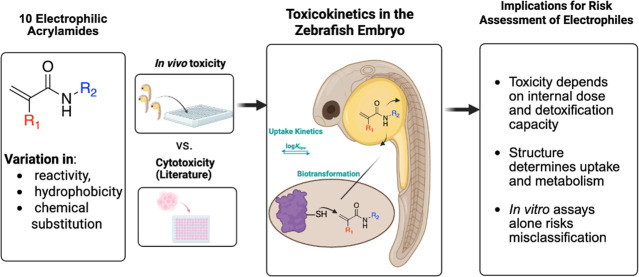

## 1 Introduction

Monomeric acrylamide is a water-soluble electrophilic compound used widely in polymer synthesis and paper production.^1,2^ Due to its high solubility and mobility, it can readily contaminate surface and groundwater.^3^ Beyond industrial sources, acrylamide is also formed during the Maillard reaction in thermally processed food and through tobacco smoke pyrolysis.^4^

Acrylamide is classified as a probable human carcinogen (IARC, Group 2A) and is known to induce neurotoxicity, developmental toxicity, and genotoxicity in various species,^5,6^ including humans.^7^ It binds covalently with biological nucleophiles such as proteins and DNA via Michael-type addition at its electrophilic β-carbon.^8^ A central target is glutathione, which serves as a major cellular defense system against oxidative stress. Glutathione conjugation of acrylamide represents a key detoxification route but can also lead to glutathione depletion and oxidative stress.^9^

While acrylamide itself has been studied in detail, far less is known about its numerous N-substituted analogues. These structural derivatives, characterized by chemical modifications at the amide nitrogen and α-carbon, are widely used in the polymer industry.^1,10,11^ The European Chemicals Agency (ECHA) lists 41 acrylamide derivatives, many of which exceed 1000 tons per year in use.^12,13^ Given this tonnage and diversity of use, potential environmental release into aquatic systems is likely, but systematic monitoring data are still lacking. From a regulatory perspective, this creates uncertainty regarding the environmental fate and effects of structurally similar, yet chemically diverse, compounds.

Chemical substitution can affect key molecular properties, especially hydrophobicity and electrophilic reactivity,^14,15^ two parameters that strongly influence a compound’s toxicokinetic (TK) and toxicodynamic (TD) behavior. In this context, TK refers to the uptake, distribution, biotransformation and elimination processes that determine the internal concentration in the ZFE whereas TD describes interactions with biological targets that translate internal dose into effects. *In vitro* studies suggest that certain N-substituted acrylamides show enhanced reactivity toward glutathione and increased cytotoxicity compared to acrylamide.^16^ However, whether these trends are maintained *in vivo*, where toxicokinetics play an important role,^17^ is largely unknown.

Biotransformation plays a dual role in toxicity modulation: it can detoxify electrophiles through conjugation (e.g., with glutathione, cysteine, or *N*-acetylcysteine), or lead to bioactivation via reactive intermediates such as epoxides. In mammals, acrylamide is transformed by CYP2E1 into glycidamide and detoxified through glutathione conjugation.^18^ A study by Tanii and Hashimoto showed that acrylamide and nine of its analogues undergo oxidative epoxidation and conjugation reactions in mouse liver microsomes.^19^ Epoxide formation is of particular toxicological relevance because such intermediates can form covalent DNA adducts and thereby contribute to genotoxicity and carcinogenicity.^20,21^ However, the biotransformation capacity of aquatic organisms, including their potential for epoxide formation, remains unclear.

Recent work in the field of New Approach Methodologies for Chemical Risk Assessment (NAMs) has emphasized the need to move beyond nominal exposure concentrations and consider internal concentrations and biotransformation to improve toxicity predictions.^22^ In this context, the zebrafish embryo (ZFE) has emerged as a promising NAM that bridges the gap between simple *in vitro* systems and complex vertebrate models.^23^ Many biotransformation enzymes are already expressed in embryonic stages, and the high genetic homology to humans, and compatibility with higher throughput testing, ZFEs offer a relevant model for toxicokinetic and toxicodynamic studies. However, little is known about the biotransformation of acrylamide analogues in this model, and on how chemical structure influences their biotransformation, internal concentrations and observed toxicity.

This study aimed to systematically investigate the role of chemical structure in modulating the toxicity, internal concentrations, and biotransformation of ten (meth)­acrylamides in the ZFE model. Specifically, we selected seven acrylamides and three methacrylamides that differ in hydrophobicity, electrophilic reactivity, and *N*-substitution. Using a combination of acute toxicity assays, internal concentration measurements, and untargeted high-resolution mass spectrometry (UPLC-QTOF-HRMS), we assessed: (1) whether (meth)­acrylamide toxicity in ZFEs is primarily determined by hydrophobicity, electrophilic reactivity, internal concentrations or biotransformation; (2) how internal concentrations correlate with physicochemical parameters, reactivity and toxicity; (3) to what extent structural features (e.g., α-methylation, *N*-substitution) determine biotransformation pathways and their importance in the ZFE and (4) whether specific biotransformation pathways modulate internal concentrations and toxicity. The present study further aims to improve the mechanistic understanding of reactive toxicants in aquatic organisms and support the development of predictive models within the context of NAMs and regulatory chemical safety assessment.

## 2 Materials and Methods

### 2.1 Chemicals, Reagents, Standards

All (meth)­acrylamides were of analytical grade. Information on specific quality grades and suppliers are provided in the Supporting Information 2 (Table S1). Ultrapure water was generated using a Merck Milli-Q Integral 5 system (Merck, Darmstadt, Germany). Chemical stock solutions were prepared by dissolving each chemical in pure methanol and stored at −20 °C. The concentration of the stock solutions were 1–250 mg/mL depending on the respective exposure concentration. The stock solutions were used for both chemical exposure and as analytical standards for quantification of the parental compounds. Detailed information on preparation of exposure media and calibration standards are provided in the Supporting Information (Tables S1 and S1). Physicochemical properties and exposure concentrations are provided in Table 1.

**Table 1:**
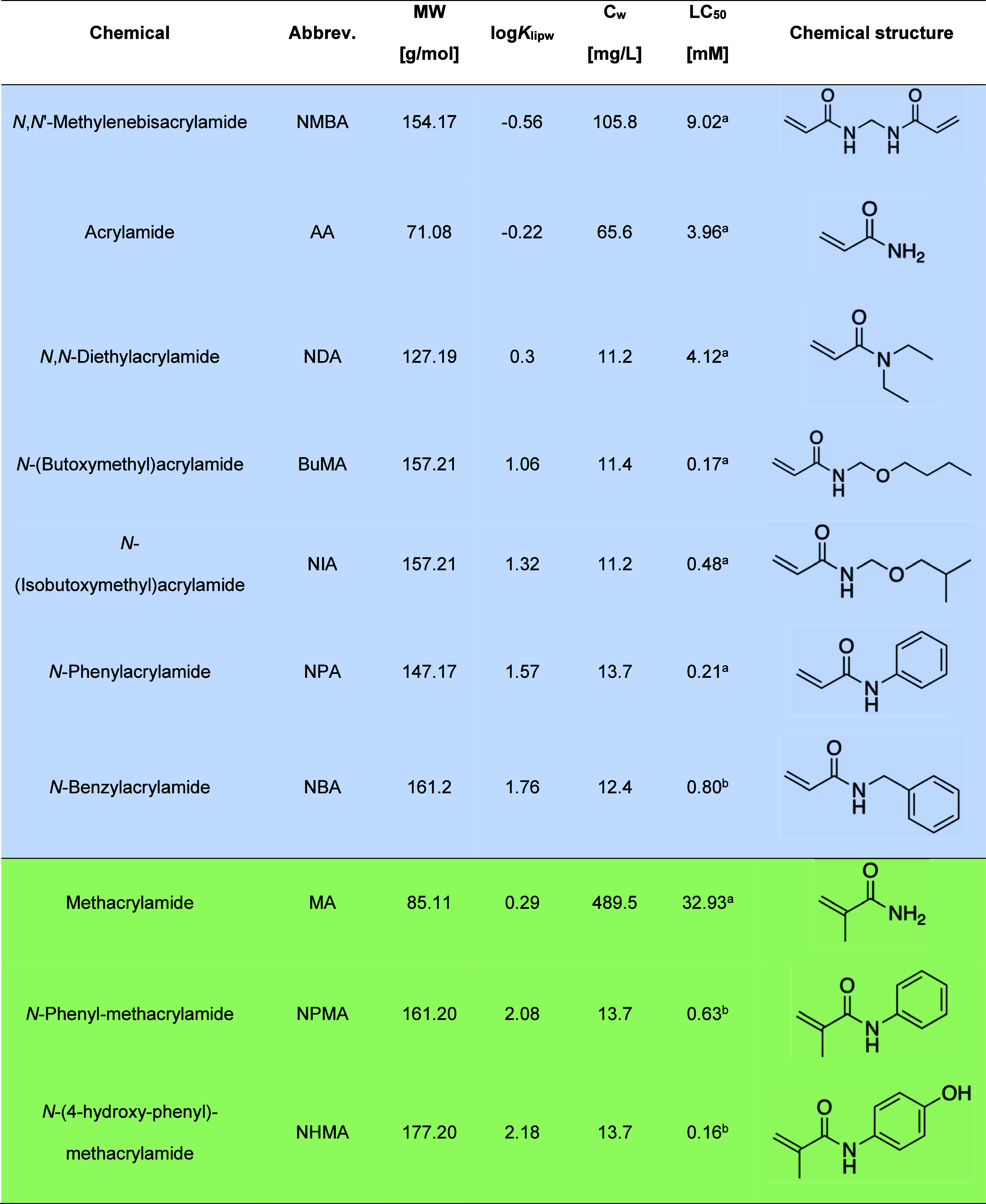
Physicochemical Properties, Compound Abbreviations, Measured Exposure Concentrations, Half-Maximal Lethal Concentrations (LC_50_) and Chemical Structures of 7 Acrylamides (Blue) and 3 Methacrylamides (Green)

a Chemicals are ordered according to their log*K*_lipw_ (pH 7.4) values. *C*_w_ – concentration in the exposure medium Grasse et al. 2024.^24^

b Determined in present study.

### 2.2 Liposome-Water Partition Coefficients

The hydrophobicity of a molecule is a crucial parameter for its uptake behavior into a cell and, therefore, vital to know to interpret observed toxicity.^25^ Since all study compounds are neutral at the ambient pH, measured liposome-water partition coefficients (*K*_lipw_) were collated from Huchthausen et al. 2023.^16^ Details on partition coefficients are described in the Supporting Information 2, (Table S1).

### 2.3 Exposure of Zebrafish Embryos

#### 2.3.1 Fish Embryo Toxicity Test

The fish husbandry was conducted according to Grasse et al. 2024.^24^ All procedures involving the care and handling of zebrafish (*Danio rerio*) were conducted in compliance with established guidelines and regulations. Approval was granted by the local government authority (Landesdirektion Sachsen, Geschäftszeichen 24-5131/252/7) ensuring adherence to ethical and legal standards. Acute toxicity data (LC_50_ values) for AA, NMBA, NDA, BuMA, NIA and MA were adapted from Grasse et al.,^24^ while data for NBA, NPA, NPMA and NHMA were determined in this study. Briefly, 35 zebrafish were held in 14 L tanks with a female-to-male ratio of 1:1 and a light–dark rhythm of 14:10 h at a constant temperature of 26 ± 1 °C. The water quality was checked biweekly by determination of pH, water hardness, conductivity, nitrate, nitrite, ammonia and oxygen saturation. After light-induced spawning, the eggs were collected in glass trays covered with a 3 mm mesh and artificial plants and rinsed with ISO water. At a developmental stage of 1 h post fertilization (hpf), four-cell stage ZFEs were selected according to Kimmel (1995)^26^ and exposure experiments were started around 4 hpf. ZFEs were exposed to the individual chemicals for 4 days on 96-well plates in aqueous media according to DIN EN ISO 7346-3 (1997) (ISO water), containing 10 mM of (4-(2-hydroxyethyl)-1-piperazineethanesulfonic acid) buffer at pH 7.4. The composition of ISO water and the calculation of neutral fractions of the test chemicals are provided in the (Supporting Information 1, Table S2 and Supporting Information 2, Table S1). Mortality was indicated by coagulation of embryos or necrosis and recorded every 24 h. Each concentration was tested in at least two independent replicates, using 16 embryos per replicate (either one embryo per well in 96-well plates).

LC_50_ values were derived from nominal aqueous concentrations (C_w_) using a log–logistic model using the R-package drc. Full dose–response curves and model diagnostics are provided in Supporting Information 2 (Sheet S12).

#### 2.3.2 Exposure Experiments in ZFEs for Biotransformation and Internal Concentration Determination

Exposure experiments were performed using the workflow described in Grasse et al. 2024.^24^ The exposure concentrations (10–250 mg/L) were selected to be 10–100 times lower than the respective measured LC_50_ value to avoid any confounding effects due to toxicity. ZFEs were exposed separately to 7 acrylamides and 3 methacrylamides. For internal concentration analysis, 24 ZFEs were exposed to the respective chemical providing three replicates per chemical (8 ZFEs/replicate). To increase the transformation products (TP) concentrations for suspect and nontarget screening, 35 ZFEs per chemical were exposed to the individual chemicals. Unexposed ZFEs were sampled as well in triplicates, serving as the negative control. The exposure was performed for 4 days at 26 ± 1 °C. Samples of ZFE extracts and exposure media were stored at −80 °C until further processing.

#### 2.3.3 Confirmation of Stability of (meth)­acrylamides in Exposure Media

The concentrations of (meth)­acrylamides were determined in ZFE media without ZFEs after 4 days to monitor any bias by chemical degradation. The incubation was performed under the same condition as in the ZFE exposure experiments. For chemical analysis, all samples were diluted with ultrapure water to the respective calibration range of each test chemicals and stored at −20 °C until targeted LC–MS/MS measurement (see Section 2.5). All 10 study chemicals showed recoveries of between 99 and 127% after a 4 days exposure at 26 ± 1 °C with a light–dark rhythm of 14:10 h indicating no significant chemical degradation or sorption loss (see Table S5 in the Supporting Information 1).

### 2.4 Sample Preparation of ZFE Extracts for Chemical Analysis

Exposed and control ZFEs were collected in FastPrep tubes filled with glass beads for methanolic analyte extraction (8 ZFEs per replicate) as described in Grasse et al.^24^ Since all ZFEs were hatched at 96 hpf, no dechorionation was required. Exposure media were removed and the ZFEs were washed three times with ISO water within 5 min. After discarding all remaining liquid, ZFEs were snap-frozen with liquid nitrogen and stored at −80 °C until further processing. For extraction of parental compounds and their TPs, 200 μL of methanol was added to each sample and ZFEs were homogenized via a FastPrep homogenizer (MP Biomedicals, USA) for 20 s at 6.5 m/s according to Halbach et al.^27^ Subsequently, the samples were sonicated for 30 min at room temperature. After centrifugation (13,000 rpm, 4 °C, 15 min), 150 μL of the supernatants were transferred into HPLC vials and diluted 1:1 (v/v) with ultrapure water. The chemical extracts were stored at −20 °C until LC–MS/MS and UPLC-QTOF-MS measurements.

### 2.5 Targeted Quantification of (meth)­acrylamides

Internal concentrations of the 10 study chemicals were determined on a 1290 Infinity HPLC system (Agilent Technologies, Böblingen, Germany) coupled to a Qtrap 5500 triple-quadrupole mass spectrometer (AB Sciex, Darmstadt, Germany) equipped with a Turbo-Ion-Spray interface. The chromatographic separation of the analytes was performed using an Atlantis T3 C_18_-phase column (2.1 mm × 50 mm, 3 μm; Waters, Eschborn, Germany) equipped with an Atlantis T3 Security Guard column (2.1 × 10 mm, Waters, Eschborn, Germany). Targeted analysis was performed using scheduled multiple-reaction-monitoring (MRM) mode.

MRM parameters can be found in Table S7 in the Supporting Information 1. For LC–MS/MS analysis, methanolic ZFE extracts were diluted to the calibration range of the analytes. The samples contained a water–methanol ratio of 1:1 (v/v) for HPLC-MS/MS analysis. The instrument was controlled by Analyst (version 1.5.2, AB Sciex). Calibration curves were obtained using a 1/x weighted regression. Detailed instrumental parameters can be found in Table S6 (Supporting Information 1) in the Supporting Information and in Grasse et al.^24^

### 2.6 UPLC-HRMS Analysis and TP Identification

The determination and identification of TPs was performed on a UPLC-QTOF-MS as described elsewhere (Grasse et al.,^24,28^ Huchthausen et al.^16^). Detailed instrumental and software parameters are provided in Grasse et al. (2024)^24^ and the Supporting Information 1 (Tables S9 and S10).

After data acquisition, TPs were tentatively identified via suspect and nontarget-screening. For suspect-screening, predicted TPs of (meth)­acrylamides (Supporting Information 2, Table S16) provided by Biotransformer 3.0 ^29^ (version 3.1.0) and experimentally determined TPs^18^ of AA were used. Predicted TPs are provided in Table S13 in the Supporting Information 2. For nontarget screening, data from ZFE extracts exposed to individual chemicals were compared to samples of unexposed ZFEs to identify newly occurring signals in the exposed sample compared to the negative control. Data analysis was conducted according to the workflow described in Grasse et al.^28^ using MarkerLynx (Waters, version 4.1). TP peak areas were used for semiquantitative comparisons only. Absolute TP abundances are uncertain because ionization efficiencies and fragmentation can vary across TPs. In the absence of authentic analytical TP standards, we were constrained to performing only qualitative data interpretations.

### 2.7 Specificity Assessment

#### 2.7.1 Toxic Ratio Analysis

The specificity of a toxic effect induced by a chemical was assessed by the toxic ratio (TR) calculated by division of the predicted LC_50_ for unspecific baseline toxicity (eq 1) and the measured aqueous LC_50_ (eq 2).^30^ A TR higher than 10 indicates an excess toxicity of a chemical relative to baseline toxicity.^30,31^ This excess toxicity indicates a potential specific or reactive mode of action, which is relevant to classify (meth)­acrylamides.

1 $$LC_{50,baseline\;toxicity}=10^{\left(-0.99\times \log{\;K}_{lipw}-2.22\right)}$$

2 $$TR=\frac{LC_{50,baseline\;toxicity}}{{LC}_{50,experimental}}$$

### 2.8 Data Processing and Statistical Analysis

#### 2.8.1 Processing of UPLC-HR-MS Data

Data processing of UPLC-HR-MS data are described in Grasse et al.^24^ in detail. Software parameters are provided in Table S10 in Supporting Information 1. In brief, TP candidates were identified by comparing exposed and control samples using MarkerLynx (Waters, version 4.1), applying retention-time and exact-mass filters (±0.1 min, ±0.01 Da), and evaluating new appearing peaks within the expected mass range (50–1200 *m*/*z*) and mass defect of the parent compound. Diagnostic fragment ions were then used to confirm plausible structural transformations (e.g., oxidation, conjugation, or hydrolysis). For semiquantification of the TPs, positive and negative ionization modes were considered separately, due to differences in ionization efficiencies of certain compounds.

#### 2.8.2 Hierarchical Clustering of TP Pathways

Hierarchical clustering analyses were performed to explore similarities among (meth)­acrylamide derivatives based on their transformation behavior.

All data processing and visualizations were performed in R (version 4.0.0) using the ComplexHeatmap, cluster, ggplot2, and RColorBrewer packages. Two distinct data domains were analyzed: peak areas of TPs divided by the number of the ZFEs used in the respective experiment. First, TPs were categorized into distinct transformation pathways. This categorization was conducted based on similarities of chemical modifications of both *N*-substituents (e.g., hydroxylation, glucuronidation, sulfation) and (meth)­acrylamide moieties (e.g., glutathione and mercapturic acid conjugates). Then, peak area data for TPs were log-transformed using the natural logarithm with an offset (log1p) to minimize skewness and z-scaled to unit variance. Compounds were clustered based on Euclidean distance using Ward’s minimum variance method (ward.D2).^32^ The optimal number of clusters (*k* = 2–6) was determined by maximizing the average silhouette width. Heatmaps were generated with the ComplexHeatmap package in R studio (version 4.0.0), with clustering applied only to parent compounds. Transformation pathways were not clustered to preserve the sequential structure of pathway development.

## 3 Results & Discussion

### 3.1 (Meth)­acrylamide Toxicity in ZFE is Determined by Reactivity and Hydrophobicity

For nonspecific toxicants without reactive properties, toxicity typically correlates linearly with hydrophobicity.^33^ Reactive compounds like acrylamide-derivatives may cause toxicity below baseline levels through covalent modification of cellular nucleophiles via Michael addition, particularly at cysteine residues and glutathione, depending on dose and exposure duration.^34^ Acute toxicity was chosen as an end point because the cytotoxicity of acrylamides strongly correlates with their glutathione reactivity, reflecting toxicity driven primarily by electrophilic reactivity and covalent protein binding.^16^ It therefore serves as a suitable integrative measure of these mechanistic processes. Unless stated otherwise, toxicity metrics refer to LC_50_ values based on nominal exposure concentrations. Nominal and measured exposure concentrations were provided in Table S5 in Supporting Information 1. To test the contribution of hydrophobicity on (meth)­acrylamide toxicity, TRs were calculated by dividing predicted baseline toxicity by measured LC_50_ values (Figure 1A). TRs between 0.1 and 10 indicate baseline toxicity, while TRs > 10 indicate a specific mode of action.^31,33^ Methacrylamides MA, NPMA, and NHMA, along with NBA, displayed TRs of 2.6, 2.3, 7.1, and 3.8, indicating baseline toxicity. Conversely, AA, NMBA, BuMA, NIA, NDA, and NPA showed excess toxicity, with TRs ranging from 17 (NIA) to 86 (BuMA), consistent with earlier findings.^16,24^

**Figure 1:**
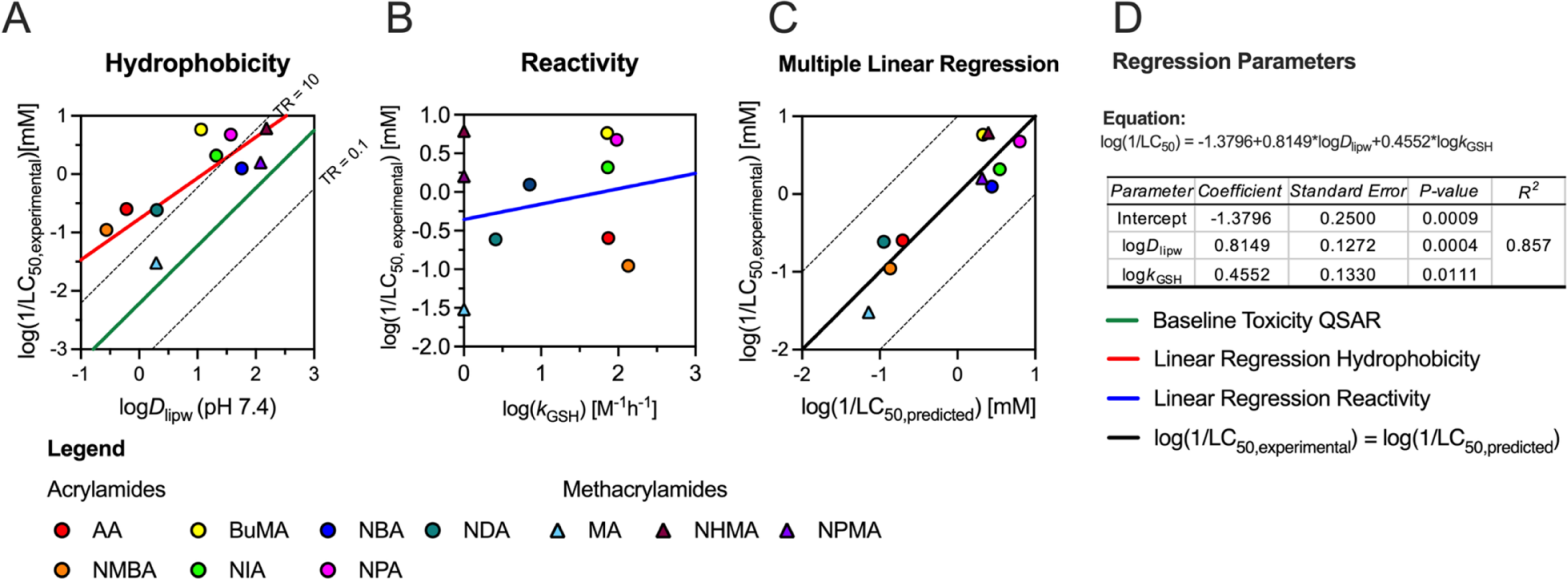
(Meth)­acrylamide toxicity in relation to hydrophobicity and reactivity against glutathione. (A) Contribution of hydrophobicity. Specificity assessment of toxicity in the ZFE, plotting experimental LC_50_ values against the lipidome water distribution ratio (log*K*_lipw_ at pH 7.4). Toxic ratios (TRs) are visualized using the baseline toxicity QSAR by Klüver et al. (green line). The dotted lines show a factor of 10 variation centered on the QSAR predictions. Linear regression between experimental LC_50_ and log*K*_lipw_ is displayed as red line. (B) Contribution of reactivity on (meth)­acrylamide toxicity in the ZFE. Blue line displays linear regression between pseudo-first-order reactivity constant (*k*_GSH_) and experimental LC_50_. (C) Comparison of experimental with predicted LC_50_ taking into account both hydrophobicity and reactivity. (D) Regression parameters of multiple linear regression. For reactivity assessments, *k*_GSH_ for MA, NPMA and NHMA were set close to 0 (1 × 10^–8^ M^–1^h^–1^) due to lack of observed glutathione reactivity.

Although the number of ten compounds is limiting a correlation analysis, the data indicate at least a weak but significant correlation between log­(1/LC_50_) and log*K*_lipw_ for (meth)­acrylamides (*R* ^2^ = 0.73, *p* = 0.0016; Figure 1A). Despite that six of the tested compounds can be considered as reactive based on their TR, glutathione reactivity (log*k*_GSH_) did not correlate with toxicity (*R* ^2^ = 0.037, *p* = 0.67; Figure 1B). However, multiple regression analysis combining log*K*_lipw_ and log*k*_GSH_ revealed a stronger correlation (*R* ^2^ = 0.87) between predicted and experimentally determined LC_50_ values, suggesting that both hydrophobicity and reactivity contribute to toxicity (Figure 1C,D). Similar trends were observed by Böhme and co-workers in *Tetrahymena pyriformis*.^15^ Despite their biological differences, both ZFEs and *T. pyriformis* rely on passive diffusion for chemical uptake. While correlations between hydrophobicity/reactivity and toxicity are mechanistically plausible, the limited chemical domain (*n* = 10) constrains statistical power. Reported correlations should therefore be considered indicative rather than confirmatory.

### 3.2 Comparison of Toxicity of (meth)­acrylamide *In Vivo* ZFE with *In Vitro* Cell Bioassay

Building on the observed correlation between hydrophobicity, reactivity and toxicity in the ZFE model, we next investigated whether similar trends are also evident in cellular systems. To this end, LC_50_ values from the ZFE were compared with IC_10_ data from three *in vitro* bioassays previously reported by Huchthausen et al. (Figure 2).^16^ In addition, the test concentrations used in the present study are not environmentally relevant, but they are suitable for elucidating structure–activity and biotransformation–toxicity relationships under controlled exposure conditions.

**Figure 2:**
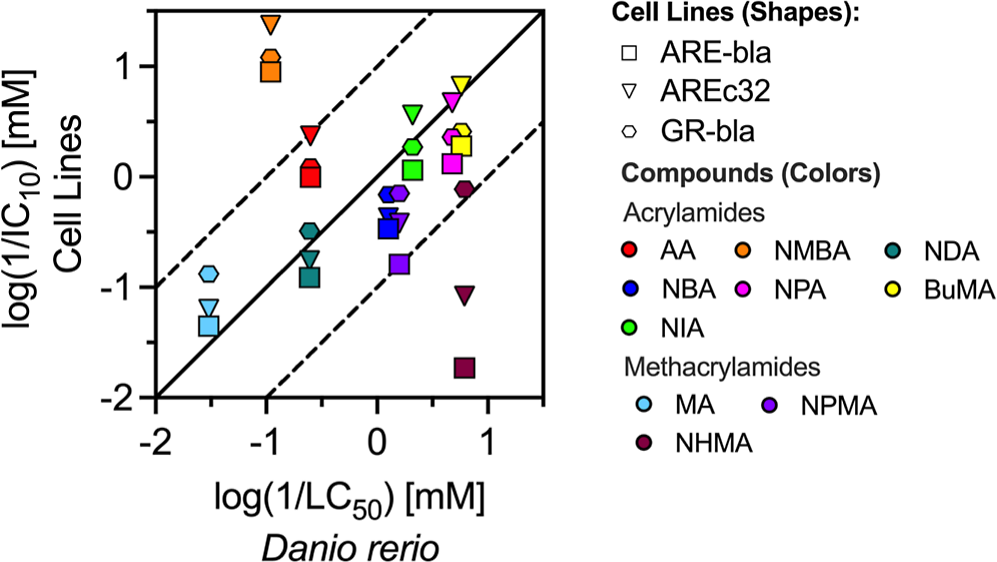
Comparison of LC_50_ values determined for the ZFE with IC_10_ values from three cell lines with different metabolic profiles determined by *in vitro* bioassays. GR-bla is based on a HEK293T cell line, ARE-bla is based on a HepG2 cell line, and AREc32 is based on an MCF7 cell line. GR-bla lacks cytochrome P450 activity, ARE-bla has higher basal CYP1 levels, and CYP1 is inducible in AREc32. ARE-bla and AREc32 also report oxidative stress via the Nrf2/Keap1 pathway, making them suitable for studying acrylamide metabolism and related cytotoxicity mechanisms. IC_10_ data were obtained from the literature.^16^

The comparison between ZFEs and human cell-based assays was conducted to illustrate how toxicokinetic factors modulate apparent potency between *in vitro* and *in vivo* systems. Both models represent waterborne exposure systems and are expected to respond similarly to reactive compounds, with differences primarily arising from toxicokinetics. Human cytotoxicity data were selected because they were available for the same compounds and obtained using standardized procedures. Acute toxicity was chosen as an end point for reactive toxicity because Huchthausen et al. demonstrated a direct linear correlation between cytotoxicity and reactivity against GSH.^16^ Human cell lines were preferred for this comparison because currently available zebrafish cell lines are not well characterized in terms of their biotransformation capacity and oxidative stress response mechanisms.

The *in vivo* LC_50_ and the *in vitro* IC_10_ values were generally within 1 order of magnitude of each other. However, NMBA (log*K*_*l*ipw_ = −0.56, *k*_GSH_ = 134 M^–1^ h^–1^) and AA (log*K*_lipw_ = −0.22, *k*_GSH_ = 74.34 M^–1^ h^–1^), despite their high reactivity, were up to 212-fold less toxic in the ZFE than in the AREc32 cell line (Figure 2). This discrepancy is likely attributed to rapid biotransformation in the ZFE—less pronounced in the studied cell lines—and may further contribute to the lower *in vivo* toxicity observed compared to *in vitro* results, which will be discussed in detail in the following section.

In contrast, NHMA (log*K*_lipw_ = 2.18) was 333-fold more toxic in the ZFE than in the ARE-bla assay, and showed 73-fold and 8-fold higher toxicity compared to AREc32 and GR-bla, respectively. However, similar compounds with comparable hydrophobicity and reactivity (e.g., NPMA), exhibited much smaller differences in toxicity between *in vitro* and *in vivo* systems, indicating that additional factors beyond hydrophobicity, such as active uptake or biotransformation, may play a role in NHMA’s higher *in vivo* toxicity in ZFE. Despite NPMA’s and NHMA’s structural similarity, differences in hydroxyl substitution on the aromatic ring may influence their transformation in ZFE. While electrophilic reactivity is a major determinant of acute cytotoxicity, compound-specific bioactivation pathways - such as formation of quinone or quinone–imine intermediates^28,35^ in aromatic acrylamides like NHMA—may contribute to different toxicity. These pathways could lead to targeted protein binding analogous to acetaminophen and warrant further mechanistic investigation.

Although hydrophobicity of (meth)­acrylamides appear to be one key driver of toxicity in the ZFE, toxicodynamic factors—such as electrophilicity—play a more important role *in vitro*. This is supported by the observation that cytotoxicity in the *in vitro* bioassays correlates with chemical reactivity toward glutathione rather than with hydrophobicity.^16^ This is consistent with findings by Chan et al., who reported a direct relationship between glutathione reactivity and *in vitro* hepatotoxicity for α,β-unsaturated carbonyl compounds.^200^ However, such trends were not observed in mice, again pointing to the critical role of toxicokinetics in modulating the biologically active dose.

Together, these findings emphasize the importance of toxicokinetics when interpreting the toxicity of (meth)­acrylamides in organisms. To further elucidate the role of toxicokinetics on *in vivo* toxicity of (meth)­acrylamides, we next investigated internal concentrations and biotransformation in the ZFE model, and how these processes affect observed toxicity.

### 3.3 Impact of Internal Concentrations on (meth)­acrylamide Toxicity in ZFE

The internal concentration is a critical factor determining toxicity. To investigate how uptake of (meth)­acrylamides influences their internal concentration, we quantified internal concentrations (C_int_) of nine of ten selected (meth)­acrylamides (except AA) in ZFEs after 4 days of exposure. AA concentrations could not be quantified in the ZFE matrix with sufficient precision because of pronounced matrix effects (>1000%) from coelution of ionization-enhancing matrix compounds.

In Figure 3A, internal concentrations were converted into BCFs by dividing internal by the external concentration. BCF values ranged from 0.06 (±0.01, NMBA) to 1.3 (±0.3, NPA), which is in line with BCFs of compounds with similar log*K*_lipw_ values.^17,24^ A significant positive correlation between log*K*_lipw_ and BCF (*R* ^2^ = 0.59, *p* = 0.0092) was observed, consistent with previous studies for neutral compounds with similar hydrophobicity.^24,33,36,37^

**Figure 3:**
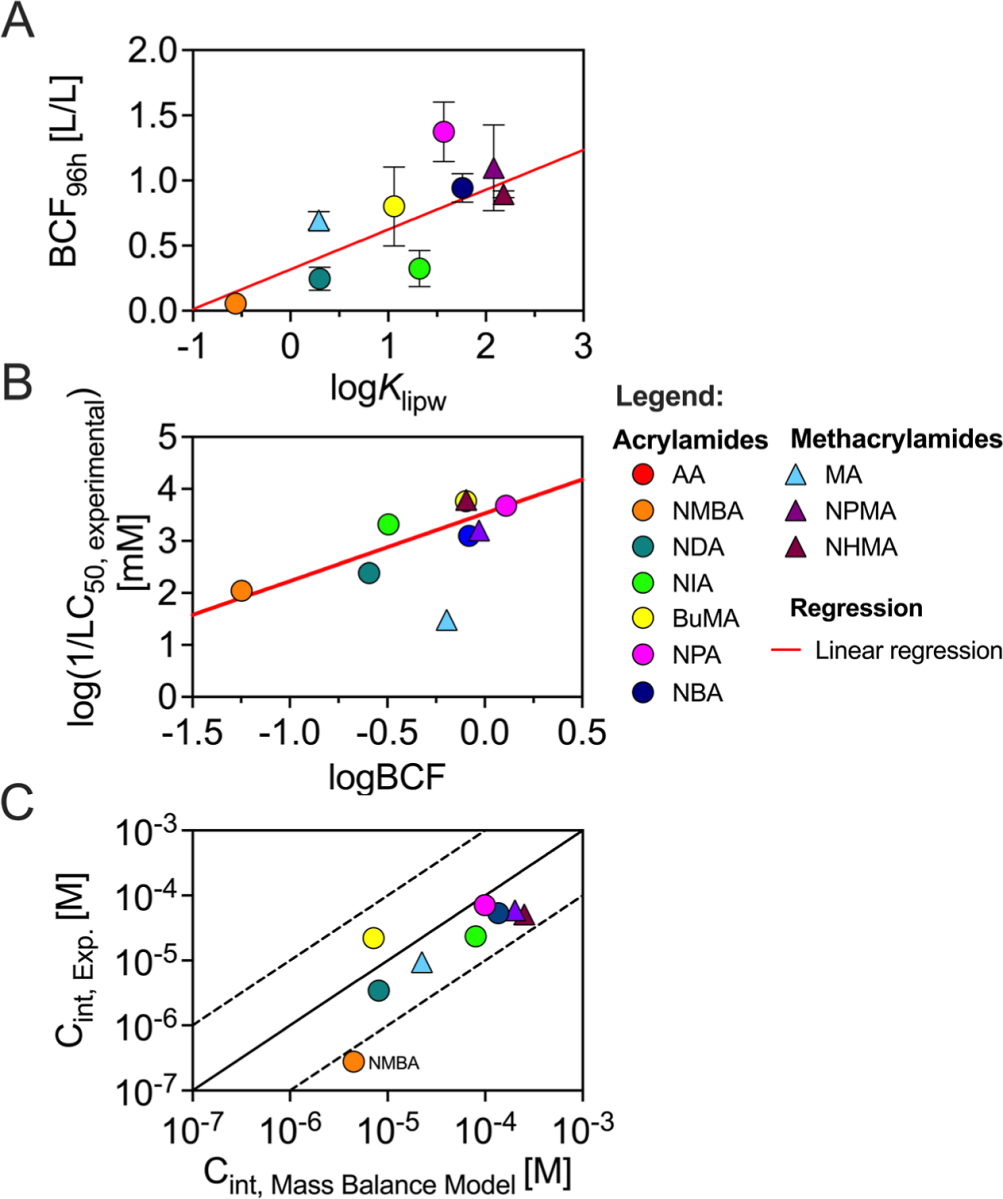
Role of hydrophobicity and internal concentrations in (meth)­acrylamide toxicity. (A) Correlation between hydrophobicity (log*K*_lipw_) and experimentally determined bioconcentration factors (BCF) in ZFEs after 96 h exposure. (B) Comparison of measured BCFs at sublethal exposures with observed LC_50_ values. (C) Comparison of predicted with measured internal concentrations. The black solid line indicates the 1:1 agreement between predicted and experimental internal concentrations, while the dotted lines represent a ±10-fold range.

We next related observed LC_50_ values against experimental BCFs in Figure 3B. For eight out of nine compounds, acute toxicity correlated with BCF (*R* ^2^ = 0.73, *p* = 3.6 × 10^–3^). However, MA deviated from this trend with relatively low toxicity (LC_50_ = 33 mM). Although MA followed the expected relationship between hydrophobicity and internal concentration (Figure 3A), the low toxicity compared to other compounds may be explained by its high polarity (log*K*_lipw_ = 0.29) and lack of electrophilic reactivity.

An additional factor influencing (meth)­acrylamide toxicity is the potential modulation of internal concentrations by biological processes. A preliminary assessment of this can be made by comparing experimental internal concentrations with predicted values derived solely from hydrophobicity, molecular weight, and charge state (Figure 3C).^24,25^ For eight of the nine compounds tested, experimental and predicted internal concentrations were in reasonable agreement. However, NMBA exhibited a 16-fold lower internal concentration than predicted (Figure 3C). Biotransformation may reduce its internal concentration, potentially explaining differences between *in vitro* and *in vivo* toxicity data from Figure 2. Given the potential of biotransformation to modulate internal concentrations, we next characterized transformation behavior in detail.

### 3.4 Impact of Biotransformation on (meth)­acrylamide Toxicity in ZFE

For reactive compounds, biotransformation can neutralize electrophilic sites and thereby reduce toxicity or lead to bioactivation via reactive intermediates. (Meth)­acrylamides are known to undergo metabolic activation to reactive epoxides such as glycidamide, which are more toxic than their parent compounds. However, such bioactivation pathways have not yet been characterized in aquatic species.^38,39^ Investigating structure-specific biotransformation in the ZFE may therefore provide critical mechanistic insight into both detoxification and potential bioactivation.

To address this, we performed a comprehensive biotransformation analysis using UPLC-QTOF-HRMS to elucidate how biotransformation of (meth)­acrylamides In ZFE is linked to their molecular structure. By tentative identification of TPs of the ten test compounds, we aimed to determine whether α-methylation, *N*-substitution, and polarity influence biotransformation and whether chemical substitution of biotransformation help explain differences in (meth)­acrylamide toxicity in the ZFE.

#### 3.4.1 Overview of Biotransformation Products

After 96 h of exposure, 90 TPs were detected across all (meth)­acrylamides, of which 78 were tentatively identified at confidence level 3 based on MS/MS fragmentation and retention times; 12 were assigned to level 4 (Supporting Information 2, Tables S2–S11). TPs were named according to parent compound abbreviations and their *m*/*z* values (e.g., AA_379). Further analytical details are provided in the Supporting Information 2 (Tables S2–S11).

Glutathione conjugates and mercapturic acids (cysteine and *N*-acetylcysteine conjugates) were observed for all compounds (Figure 4). In contrast to mammalian systems,^18^ no genotoxic glycidamides or corresponding nucleic acid adducts (e.g., N7-guanine-acrylamide) were detected. These TPs may be below detection limits or absent due to differences in enzyme expression. This likely reflects the absence of CYP2E1 in ZFE, the enzyme responsible for (meth)­acrylamide epoxidation in mammals.^40^

**Figure 4:**
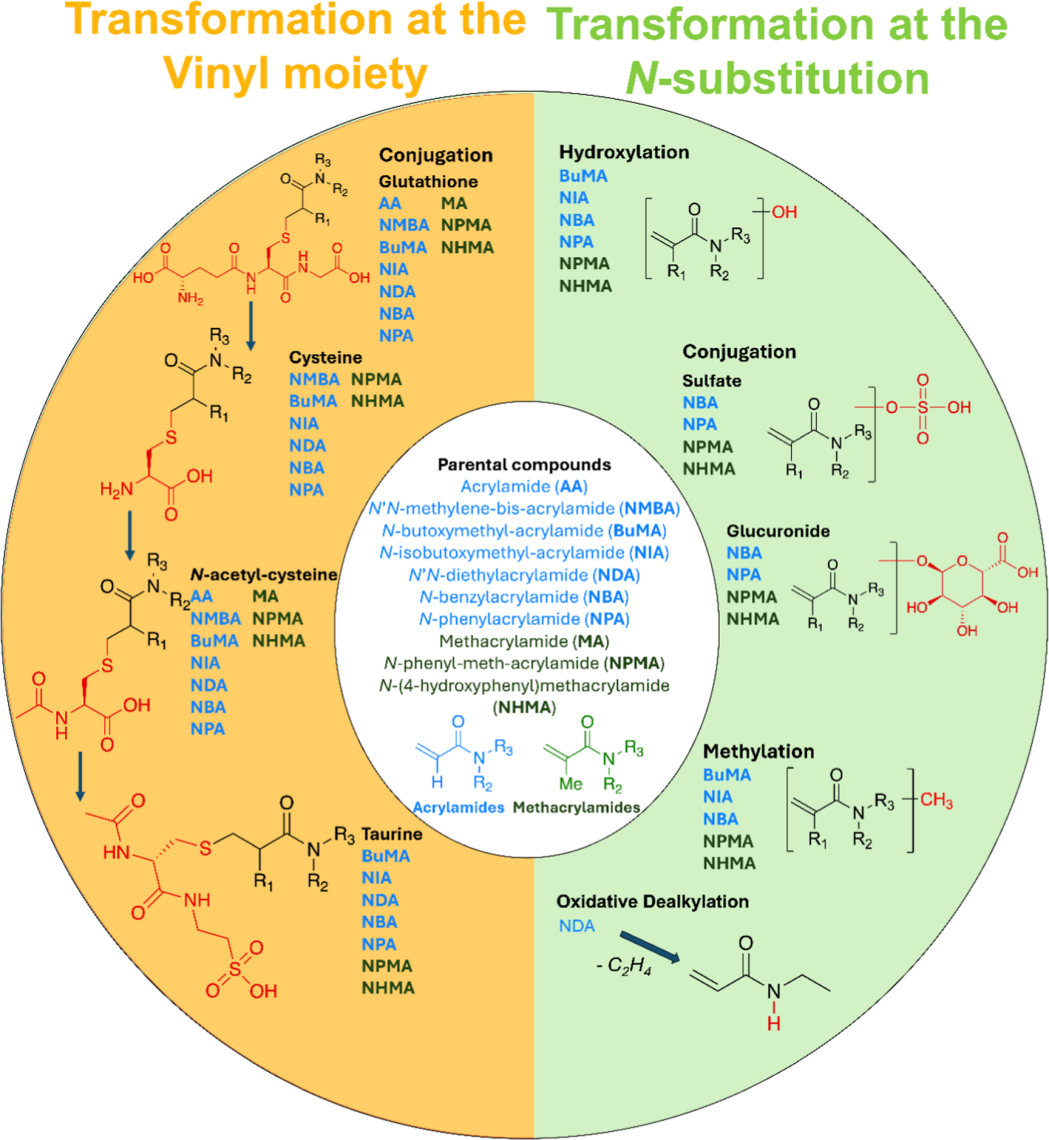
Overview of detected biotransformation products of 10 different substituted (meth)­acrylamides in extracts of exposed ZFEs. Transformations were distinguished into chemical modifications at the reactive vinyl moiety and the *N*-substituents.

Besides glutathione-related TPs, additional products of hydroxylation, sulfation, glucuronidation, methylation, and taurine conjugation were tentatively identified (Supporting Information 1, Table S11).

BuMA, NIA, and NBA showed unique reactivity by forming dimers and trimers, possibly via oxygen- or light-induced radical pathways.^14^ These oligomers were absorbed by embryos and further conjugated (e.g., with cysteine, taurine, sulfate; Supporting Information 1, Table S12). MS/MS spectra for selected oligomers of BuMA and NBA are provided in Figures S3 and S4 in Supporting Information 1. This reactivity might be attributed to electronic activation of the methylene group adjacent to the amide nitrogen and electron-withdrawing substituents. No such oligomerization was observed for the remaining compounds, highlighting structural specificity of this reaction pathway. Since exposure experiments were conducted at relatively high concentrations (10 mg/L), oligomerization is likely favored under these conditions and is probably not occurring at environmental concentrations.

To facilitate comparative assessments, TPs were grouped into (I) modifications of the reactive vinyl moiety and (II) transformations at the *N*-substituent (Figure 4). This classification enabled systematic analysis of structure–biotransformation relationships, including the impact of *N*-substitution and α-methylation, which are discussed in the following sections.

#### 3.4.2 Comparison of Acrylamide and Methacrylamide Biotransformation

Acrylamides possess an electrophilic vinyl group prone to rapid, nonspecific reactions with biological nucleophiles. In contrast, methacrylamides feature α-methylation, which reduces electrophilicity at the β-carbon via inductive and steric effects, potentially altering their biotransformation. To assess the influence of this structural difference, we compared the biotransformation profiles of acrylamides and methacrylamides and only focused on reaction at the vinyl groups (Figure 4).

Across all compounds, glutathione conjugates and mercapturic acids were detected. These findings are in agreement with literature describing this pathway as a key contributor to detoxification of acrylamide.^41^ Previous studies showed that methacrylamides do not form glutathione conjugates without enzymes,^14,16^ suggesting that the conjugation observed in the ZFE is enzyme-mediated. The presence of mercapturic acids supports this, since their formation involves several enzyme-catalyzed steps beyond direct chemical reaction.^42^

Furthermore, taurine-conjugated mercapturic acids (MAA + Tau) were tentatively identified for eight of the ten compounds, but absent in AA and NMBA, the two most polar compounds. To our knowledge, taurine conjugation of (meth)­acrylamide-derived mercapturic acids were not previously described in the literature. Since taurine conjugates occurred in both acrylamide and methacrylamide analogues, α-methylation appears not to be relevant for this pathway. Instead, uptake kinetics and more effective transformation via glutathione pathway may limit their formation.

#### 3.4.3 Influence of *N*-Substitution on Biotransformation of Different (meth)­acrylamides

To assess the influence of *N*-substitution on biotransformation, we analyzed the transformations at this site of all ten (meth)­acrylamides. Unlike transformations at the vinyl group, *N*-substituents underwent diverse reactions including phase I hydroxylation and hydrolysis as well as phase II conjugations such as sulfation, glucuronidation, and methylation (Figure 5A). These were categorized into seven distinct pathways to enable a semiquantitative cluster analysis (Figure 5). Semiquantitative analysis based on log-transformed and *z*-score normalized peak areas enabled hierarchical clustering, which revealed three main clusters based on *N*-substitution patterns (Figure 5B). Notably, ionization efficiency differences may bias intensity-based comparisons. Since the cluster analysis relies on relative differences in TP profiles and thus does not require absolute quantification, the observed patterns remain interpretable within the scope of this study due to consistent sample preparation, standardized analytical conditions, and data normalization.

**Figure 5:**
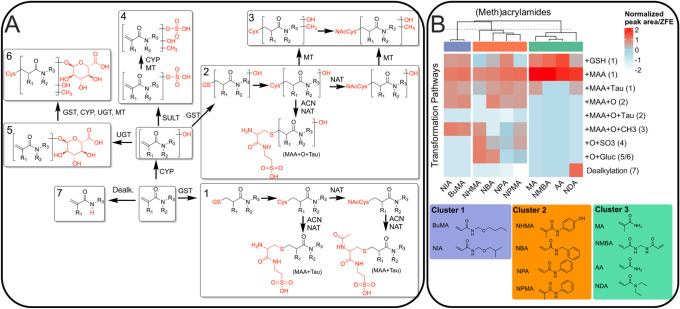
Transformation of (meth)­acrylamides in ZFEs are impacted by *N*-substitution but not β-methylation. (A) Proposed transformation pathways of different *N*-substituted (meth)­acrylamides in *Danio rerio*. Pathways were proposed based on tentatively identified TPs via HR-MS and nontarget screening approach. The ten chemicals were assigned to the pathways shown based on their detected TPs. Enzymes: CYP—Cytochrome P450, ACT—acyltransferase, UGT—Uridine 5′-diphospho-glucuronosyltransferase, SULT—sulfotransferase, NAT—*N*-acetyltransferase, MT—methyltransferase. Biomolecules: GSH—glutathione, Cys—Cysteine, NAcCys—*N*-acetylcysteine, Tau–taurine, Gluc–glucuronic acid, MAA—mercapturic acid. (B) Heatmap of log-transformed (ln­(*x* + 1)\ln­(*x* + 1) ln­(*x* + 1)) and *z*-scaled TP pathways, illustrating hierarchical clustering (Ward’s method) applied to columns to group similar biotransformation pathways. The color gradient from white to red represents increasing normalized peak areas/embryo values. Colors label respective clusters, which are listed below the heatmap. Nominal exposure concentrations were 250 mg/L (MA), 100 mg/L (AA, NMBA) and 10 mg/L (BuMA, NIA, NBA, NPA, NPMA, NHMA).

Aliphatic-substituted compounds BuMA and NIA (Cluster 1) primarily followed pathways involving phase I hydroxylation and phase II methylation (Pathways 1–3), while aromatic-substituted compounds (NBA, NPA, NPMA, NHMA; Cluster 2) predominantly underwent phase I hydroxylation followed by phase II sulfation and glucuronidation (Pathways 4 and 5). Compounds with small or no *N*-substituents (AA, MA, NMBA, NDA; Cluster 3) mainly exhibited glutathione conjugation (Pathway 1). Notably, the proposed pathways (Pathways 1–2) should be regarded as tentative, as some conjugates, such as the GSH, mercapturic acid, and taurine–cysteine adduct, could also arise from direct, nonenzymatic Michael addition rather than enzyme-catalyzed reactions.

The absence of *N*-substituent modification in NDA may be explained by steric hindrance from its ethyl groups, consistent with previous quantum-mechanical predictions.^16^ In addition, NDA showed oxidative loss of one ethyl group, which most likely corresponds to an oxidative *N*-dealkylation pathway commonly catalyzed by cytochrome P450 enzymes, rather than a hydrolytic process.^43^

Furthermore, the semiquantitative analysis revealed the glutathione/mercapturic acid pathway (Figure 5A, Pathway 1) as dominant pathway across all study compounds with 16–100% of the total peak areas, which is calculated by division of peak areas of individual TPs by the sum of all TPs of the respective compounds. To assess the relevance of this pathway on the toxicity of (meth)­acrylamides and the role of alternative pathways (2–7), we next compared the normalized peak areas obtained in negative ionization mode to both log­(1/LC_50_) and log*K*_lipw_.

#### 3.4.4 Contribution of Biotransformation Pathways to Toxicity Modulation

Gutathione conjugation followed by mercapturic acid formation appears to be a key detoxification mechanism for (meth)­acrylamides in the ZFE, particularly for polar, poorly permeable compounds. To explore its potential role in modulating toxicity of different (meth)­acrylamides in the ZFE, we compared the normalized peak areas of TPs from all pathways with observed toxicity and hydrophobicity (Figure 6). Despite the absence of quantitative validation through the utilization of analytical reference standards, the normalized TP contributions offer a semiquantitative approach to evaluate the relative significance of specific pathways, as evidenced in preceding studies.^24,28^ Consequently, the observed association between elevated relative glutathione-pathway signal and observed toxicity should be regarded as semiquantitative rather than confirmatory.

**Figure 6:**
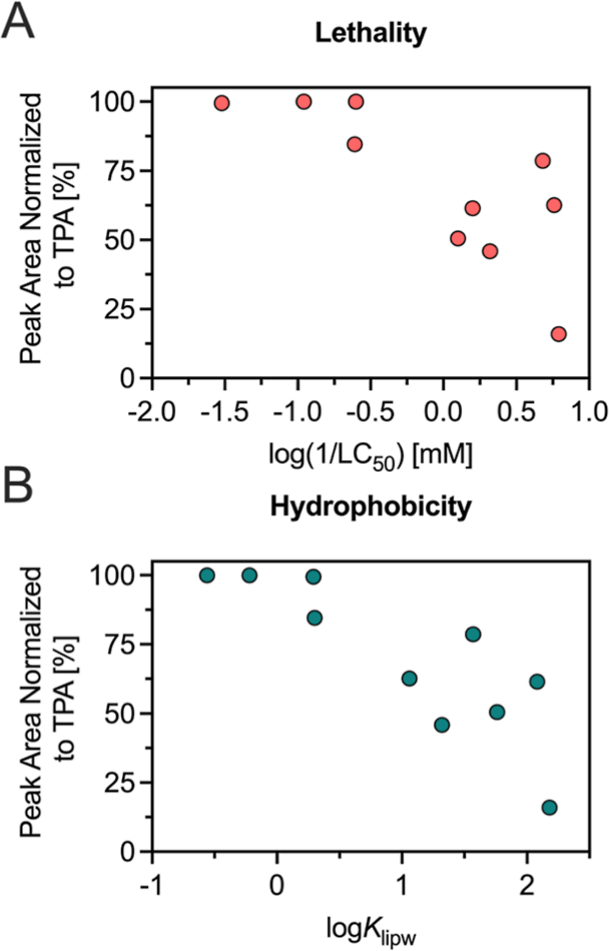
Relationship between normalized peak areas of TPs of transformation pathway 1 (glutathione and mercapturic acid conjugates) with (A) Acute toxicity (LC_50_, *R* ^2^ = 0.60, *r* = −0.77, *p* = 0.0086, *n* = 10), and with (B) Hydrophobicity (log*K*_lipw_, (log*K*_lipw_; *R* ^2^ = 0.69, *r* = −0.83, *p* = 0.003)).

A visible negative trend between the normalized peak areas of glutathione-related TPs and toxicity was observed (Figure 6A). In other words, compounds with lower relative contributions from Pathway 1 tended to be more toxic, suggesting a detoxifying role of this pathway in the ZFE, though alternative explanations, such as different contributions of other TP pathways, cannot be excluded. However, no apparent trend between toxicity and other transformations (e.g., glucuronidation, sulfation) was observed (Figure S5). This may indicate that these pathways predominantly increase polarity and promote excretion, but do not directly neutralize the electrophilic vinyl group responsible for reactivity.

A similar negative trend was observed between pathway 1 TP normalized peak areas with hydrophobicity (Figure 6B): the relative contribution of peak areas of pathway 1 related TPs decreased with increasing log*K*_lipw_, while there was no apparent trend for pathways 2–7.

When comparing across the ten study compounds, a trend emerged in which those with lower hydrophobicity (i.e., lower log*K*_lipw_) showed a higher relative contribution of glutathione-derived TPs (Pathway 1), while more hydrophobic compounds relied more on alternative transformations (e.g., sulfation or glucuronidation). This might indicate that polar (meth)­acrylamides, which exhibit limited membrane permeability, may rely more on biotransformation to modulate internal concentrations. NMBA and AA, for instance, showed both low hydrophobicity and a 100% contribution of glutathione-derived TPs to the total peak area as well as low BCF (see Figure 3). In contrast, NHMA, the most hydrophobic one, only showed 14% contribution of pathway 1 of the total peak area, while alternative pathways including sulfation (Pathway 4, 40% of total peak area) or glucuronidation (Pathway 5, 32% of total peak area) appear to be more relevant.

Taken together, these trends indicate that Pathway 1 may reflect a major detoxification pathway that is modulated by both chemical structure (especially *N*-substitution), hydrophobicity and uptake behavior. These data support the hypothesis that early and extensive glutathione conjugation, particularly of polar compounds (e.g., NMBA), may provide a mechanistic explanation for observed *in vivo*/*in vitro* discrepancies in this study. Our finding of a negative trend between normalized glutathione and mercapturic acid adduct peak areas and log­(1/LC_50_) values is in line with mechanistic data from Komoike et al., where acrylamide exposure induced upregulation of gstp1 expression^44^ and oxidative stress response. In that previous study, AA toxicity was attenuated by *N*-acetylcysteine and enhanced by GST inhibition, emphasizing the relevance of glutathione-mediated detoxification pathways. However, to establish a causal detoxification role, targeted quantification using authentic standards together with functional perturbation experiments (e.g., GST inhibition or glutathione depletion) across multiple compounds will be required to confirm pathway-specific protection.

## 4 Research Implications

This study demonstrates that the *in vivo* toxicity of (meth)­acrylamides in ZFEs is primarily determined by a combination of intrinsic potency and internal concentration, the latter being mainly governed by uptake and biotransformation. A systematic analysis of biotransformation products of ten structurally diverse monomeric (meth)­acrylamides revealed that the *N*-substitution pattern is a critical determinant of the biotransformation pathways. Conjugation to glutathione and subsequent degradation to mercapturic acids represented a conserved biotransformation pathway across all ten study compounds in ZFE. A comparison of the corresponding normalized peak areas with observed toxicity suggested a protective role of this pathway, as toxicity increased with decreasing normalized peak areas of glutathione adducts and mercapturic acids. For example, reactive but polar compounds such as NMBA showed disproportionately low toxicity *in vivo*, likely due to limited uptake and rapid transformation, while compounds with similar reactivity but higher hydrophobicity, were more up to 2 orders of magnitude more toxic *in vivo* than polar (meth)­acrylamides. Importantly, these conclusions are based on a small, intentionally diverse set of ten (meth)­acrylamides. Consequently, the observed relationships require validation across broader chemical space and with quantitative TP measurements before being generalized for regulatory use.

Nevertheless, these findings underline that uptake and biotransformation are essential determinants of *in vivo* toxicity of reactive compounds in aquatic organisms. This has direct implications for the interpretation of *in vitro* data: cell-based assays, which lack whole-organism toxicokinetics, may overestimate the hazard of electrophilic compounds that are effectively detoxified or poorly absorbed *in vivo*, leading to mechanistic misinterpretations. Practically, internal concentration and TP data from ZFEs could be directly used to improve toxicokinetic modeling and regulatory extrapolations. For example, measured BCFs and semiquantitative TP profiles can serve as input for TKTD or PBTK models to better estimate internal exposure at effect levels. In a regulatory context, such data can help identify acrylamide analogues that show strong reactivity *in vitro* but low internal concentrations and active detoxification *in vivo*—potentially avoiding false-classifications as compounds with unspecific baseline toxicity. Finally, developing quantitative TP data with authentic standards would allow these findings to be more directly incorporated into model-based risk assessments. These implications highlight that the ZFE represents a valuable *in vivo* model to resolve such ambiguities, particularly for polar and reactive compounds for which *in vitro*–*in vivo* discrepancies are most pronounced.

## Supplementary Material





## Notes

Preparation of standard and exposure solutions; devices and software; Quality assurance and quality control; Instrumental parameters; Uptake kinetics of acrylamides and MS/MS and MS^3^ spectra of distinct transformation products (PDF)
Excel file (SI2) Literature data, physicochemical properties and internal concentrations of test chemicals (S1). MS and MS^2^ Data for transformation products (S2 – S11). Dose–response-curves (S12) (XLSX)

## Abbreviations

AA: acrylamide
ACS: acyl-CoA synthetase
BCF: bioconcentration Factor
BuMA: *N*-butoxymethyl-acrylamide
Cys: cysteine
Gluc: glucuronic acid
GSH: glutathione
Hpf: hours post fertilization
MA: methacrylamide
NAcCys: *N*-acetylcysteine
NAT: *N*-acetyl transferase
NBA: *N*-benzyl acrylamide
NDA: *N*′*N*-diethyl-acrylamide
NHMA: *N*-(4-hydroxy-phenyl)-methacrylamide
NIA: *N*-isobutoxymethyl-acrylamide
NMBA: *N*′*N*-methylene-bisacrylamide
NPA: *N*-phenyl acrylamide
NPMA: *N*-phenyl-methacrylamide
OBu: butoxy-
OiBU: isobutoxy-
SR: specificity ratio
Tau: taurine
TPA: total peak area
TR: toxic ratio
ZFE: Zebrafish embryo.
