# Supplementary material for: Uptake and Biotransformation Govern the Toxicity of Reactive Acrylamides in an In Vivo Zebrafish Embryo Model: Implications for NAM-Based Hazard Assessment
Source: Environ Sci Technol. 2026 Feb 18;60(8):6546–57. doi: 10.1021/acs.est.5c10178 (PMC12961924; doi:10.1021/acs.est.5c10178)
Supplement: Supplementary file 1 [file es5c10178_si_001.pdf]

Supporting information_1 for

Uptake and Biotransformation Govern the Toxicity of Reactive Acrylamides in an In Vivo Zebrafish Embryo Model: Implications for NAM-Based Hazard Assessment

Nico Grasse[a], Stefan Scholz[b], Thorsten Reemtsma[a,c], Qiuguo Fu[a*]

[a] Department of Environmental Analytical Chemistry, Helmholtz-Centre for Environmental Research – UFZ, Permoserstrasse 15, 04318 Leipzig, Germany.

[b] Department of Ecotoxicology, Helmholtz-Centre for Environmental Research – UFZ, Permoserstrasse 15, 04318 Leipzig, Germany.

[c] Institute for Analytical Chemistry, University of Leipzig, Linnestrasse 3, 04103 Leipzig, Germany.

Corresponding author

Qiuguo Fu − Department of Environmental Analytical Chemistry, Helmholtz- Centre for Environmental Research-UFZ, 04318 Leipzig, Germany; orcid.org/0000-0002-4227-5948; Email: qiuguo.fu@ufz.de

Number of pages: 25

Number of figures: 5

Number of tables: 12

Number of equations: 2

Contents

1. MATERIALS AND METHODS……………………………………………………………S1

1.2 Solvents, buffers and additives………………………………………………………S2

1.3 Preparation of ISO water with HEPES buffer………………………………………S2

1.4 Devices and Software…………………………………………………………………S3

2. QUALITY ASSURANCE AND QUALITY CONTROL…………………………………S4

3. INSTRUMENTAL PARAMETERS……………………………………………………S5

3.1 HPLC-MS/MS analysis……………………………………………………………..S5

3.1.1 Method validation S7

3.1.2 Matrix effects and recovery S8

3.2 UPLC-QTOF-MS 9

3.2.1 Detection of transformation products (TPs) using UPLC-QTOF-MS S9

3.2.2 Identification of TPs via suspect- and non-target-screening S10

4. BIOTRANSFORMATION PRODCUTS OF (METH)ACRYLAMIDES IN THE ZFE S12

Figures

Figure 1: A Method recoveries and **B** Matrix recoveries S9

Figure S2: MS/MS fragment spectrum of BuMA conjugated to N-acetylcysteine (NAcCys) and taurine (Tau) (BuMA_426) with proposed chemical structures of fragmens S22

Figure S3: Fragment spectra of the glucuronated metabolite of NBA modified with one additional benzyl group (NBA_442).. S23

Figure S4: Fragment spectra of the glucuronated NBA metabolite (NBA_12) with two additional benzyl.. .... S24

Figure S5: Relationship between normalized peak areas of TPs of transformation pathways) with (A) Hydrophobicity ($logK_{LIPW}$) and acute toxicity (LC_{50}). S25

Tables

TABLE S1: SOLVENTS, BUFFERS AND ADDITIVES. S2
TABLE S2: COMPOSITION OF ISO WATER. S2
TABLE S3: SUMMARY OF DEVICES USED IN THIS STUDY. S3
TABLE S4: SOFTWARE WITH INFORMATION ABOUT SUPPLIER AND VERSION. S3
TABLE S5: STABILITY ASSESSMENT OF 10 STUDY CHEMICALS. ANALYTE CONCENTRATIONS WERE STARTED BEFORE AND AFTER A 96 H INCUBATION WITH AND WITHOUT ZEBRAFISH EMBRYOS S4
TABLE S6: INSTRUMENTAL PARAMETERS FOR TARGETED LC-MS ANALYSIS. S5
TABLE S7: MRM PARAMETERS OF STUDY COMPOUNDS INCLUDING RETENTION TIMES. S6
TABLE S8: LOD, LOQ AND LDR DATA FOR EACH STUDY CHEMICALS S8
TABLE S9: INSTRUMENTAL PARAMETERS OF UPLC-QTOF-MS METHOD FOR TRANSFORMATION PRODUCT ANALYSIS. S10
TABLE S10: PARAMETER OF MARKERLYNX METHOD USED FOR DETECTION OF TRANSFORMATION PRODUCTS S11
TABLE S11: SUMMARY OF (METH)ACRYLAMIDES AND RESPECTIVE BIOTRANSFORMATION PRODUCTS FORMED IN DANIO RERIO. S12
TABLE 12: UPLC-HRMS DATA AND PROPOSED IDENTITY OF DIMERS AND TRIMERS OF N-(BUTOXYMETHL)-ACRYLAMIDE (BUMA), N-(ISOBUTOXYMETHYL)-ACRYLAMIDE (NIA) AND N-BENZYL-ACRYLAMIDE (NBA) DETECTED IN ZFE EXTRACTS IN POSITIVE AND NEGATIVE IONIZATION MODES. S18

1. Materials and Methods

1.1 Chemicals and calibration standards

Acrylamide (CAS 79-06-1, AA), *N'N*-methylene-bis-acrylamide (CAS 110-26-9, NMBA), *N*-(butoxymethyl)-acrylamide (CAS 1852-16-0, BuMA), *N*,*N*-diethylacrylamide (CAS 2675-94-7, NDA) and methacrylamide (CAS 79-39-0, MA) were obtained from Sigma Aldrich (Madrid, Spain). *N*-(isobutoxymethyl)-acrylamide (CAS 16669-59-3, NIA), *N*-phenylmethacrylamide (CAS 1611-83-2, NPMA), *N*-phenylacrylamide (CAS 2210-24-4, NPA) and *N*-(4-hydroxyphenyl)methacrylamide (CAS 19243-95-9, NHMA) were purchased from TCI (Eschborn, Germany). *N*-benzylacrylamide (CAS 133304-62-6, NBA) was provided by Alfa Aesa (Haverhill, USA). Chemical structures and physicochemical properties of the ten study chemicals are shown in **Table 1**. Except NBA (96 %), all test chemicals had a purity of 98 – 100 %. Methanol and formic acid (FA) used for chemical analysis were of analytical grade and purchased from Biosolve (Valkenswaard, The Netherlands). Ultrapure water was generated using a Merck MilliQ Integral 5 system (Merck, Darmstadt, Germany). Chemical stock solutions were prepared by dissolving each chemical in pure methanol. The concentration of the stock solutions were 1 – 500 mg/mL depending on the respective exposure concentration. The stock solutions were used for both chemical exposure and as analytical standards for quantification of the parental compounds. For chemical exposure experiments, the (meth)acrylamides were dissolved in a medium defined by the International Organization for Standardization (ISO) containing 10 mM of 4-(2-hydroxyethyl)-1-piperazine-ethanesulfonic acid (HEPES) at pH 7.4 (ISO water) as described in Grasse et al. 2024[46]. Calibration standards were prepared by pooling the stock solutions to a 10 µg/mL methanol mixture of the analytes. Method-matched calibration was performed according to Grasse et al. 2024[46]. Briefly, for method-matched calibration, 8 ZFEs per sample were extracted with methanol standard solution with concentrations between 0.05 and 100 ng/mL and diluted 1:1 (v/v) with ultrapure water covering the linear dynamic ranges of all analytes.

1.2 Solvents, buffers and additives

Table S1: Solvents, buffers and additives.

Substance	CAS number	Supplier	Quality
Ammonium formate	540-69-2	Sigma-Aldrich (Munich, Germany)	>99 %
HEPES	7365-45-9	Carl Roth GmbH + Co. KG (Karlsruhe, Germany)	>99.5 %
Methanol	67-56-1	Biosolve (Valkenswaard, Netherlands).	> 99 %
Formic acid	64-18-6	Biosolve (Valkenswaard, Netherlands).	> 98 %

1.3 Preparation of ISO water with HEPES buffer

For the preparation of assay media, A solution of 10 mM (4-(2-hydroxyethyl)-1-piperazineethanesulfonic acid) (HEPES) with a pH of 7.4 in ISO water was used. The ISO water was prepared from four different salts in accordance with DIN EN ISO 7346-3 (1997).

Table S2: Composition of ISO water.

Stock solution	Ingredients	Concentration [g/L]
1	$CaCl_2 \cdot 2\ H_2O$	11.760
2	$MgSO_4 \cdot 7\ H_2O$	4.932
3	$NaHCO_3$	2.52
4	KCl	0.22

1.4 Devices and Software

Table S3: Summary of devices used in this study with model and supplier information.

Device	Supplier, model
Analytical Balance	Mettler, PM4800
Incubator	Heraeus Vötsch, Bioline VB 1514
Centrifuge	VWR, Galaxy 14 D
Ultrasonic bath	Emerson, Branson 5200
Vortexer	Heidolph REAX 2000
pH-Meter	Inolab /WTW; Knick, pH-Meter 765
Light microscope	Olympus, SZX2-ILLT
MilliQ Water System	Merck, Direct 8

Table S4: Software with information about supplier and version.

Software	Supplier, version
MassLynx	Waters, 4.1
TargetLynx	Waters, 4.1
MarkerLynx	Waters, 4.1
Analyst	AB Sciex, 1.6.2
MulitQuant	AB Sciex, 3.0.3
GraphPad Prism	10.0.2 (171)
R studio	4.0.0

2. Quality assurance and quality control

Table S5: Stability assessment of 10 study chemicals. Analyte concentrations were started before and after a 96 h incubation with and without zebrafish embryos (ZFEs) to exclude potential degradation of the chemical during the exposure experiments.

Chemical	Nominal concentration [mg/L]	Start concentration [mg/L]	Concentration after 96 h of exposure with ZFEs [mg/L]	Concentration after 96 h of exposure without ZFEs [mg/L]	Recovery [%]
AA	100	63.7	65.6	69.5	103%
NMBA	100	100.0	105.8	104.4	106%
BuMA	10	10.6	11.4	10.6	107%
NIA	10	11.3	11.2	11.0	99%
NDA	10	11.3	11.2	11.0	99%
NBA	10	9.8	12.4	11.0	127%
MA	250	479.6	489.5	462.3	102%
NPA	10	13.2	13.7	12.3	104%
NPMA	10	11.2	12.7	11.0	113%
NHMA	10	11.8	13.7	13.1	116%

3. Instrumental parameters

3.1 HPLC-MS/MS analysis

Instrumental parameters for chemical analysis are collated from Grasse et al. (2024).

Table S6: Instrumental parameters for targeted LC-MS analysis.

Parameter	Value
Ionization mode	positive
Flow rate	0.5 mL/min
Column temperature	35 °C
ionization voltage	5500 V
Source temperature	140 °C
Desolvation temperature	550 °C
Gas 1	50 psi
Gas 2	50 psi
Curtain gas	40 psi
Entrance potential	10 V
Scan cycles	600
Cycling time	1.5 sec
MRM detection window	90 sec
Target scan time	0.5 sec
Dwell time	3 – 250 ms
Mobile phase	A: MilliQ water + 0.1 % formic acid<br>B: methanol + 0.1 % formic acid
Solvent gradient	0.0 min, 10 % B; 2 min, 50 % B; 3 min, 100 % B; 6 min, 100 % B; 6.1 min, 10 % B; 15 min, 10 % B.

Table S7: MRM parameters of study compounds including retention times.

Analyte	ESI mode	Precursor m/z	Product m/z	RT [min]	DP [V]	EP [V]	CE [V]	CXP [V]
Methylacrylamide	positive	86.0	58.1	0.97	56	10	17	8
		86.0	41.1	0.97	56	10	23	6
N,N-diethylacrylamide	positive	128.0	74.1	2.61	61	10	19	8
		128.0	55.0	2.61	61	10	23	8
N,N-methylene-bisacrylamide	positive	155.0	72.2	1.29	36	10	13	6
		155.0	81.0	1.29	36	10	39	10
N-butoxymethyl-acrylamide	positive	158.03	55.0	2.97	56	10	23	8
		158.0	84.0	2.97	56	10	9	10
N-isobutoxymethyl-acrylamide	positive	158.0	55.0	2.96	56	10	25	8
		158.0	84.0	2.96	56	10	11	8
N-benzyl-acrylamide	positive	162.1	91.1	2.81	86	10	18	8
		162.1	65.1	2.81	86	10	40	10
N-phenyl-acrylamide	positive	148.0	94.0	2.90	86	10	27	16
		148.0	106.1	2.90	86	10	19	6
N-phenyl-methacrylamide	positive	162.1	134.1	3.09	86	10	14	7
		162.1	69.1	3.09	86	10	18	8
N-(hydroxyphenyl)methacrylamide	positive	178.1	150.0	2.35	86	10	14	6
		178.1	69.1	2.35	86	10	18	8

3.1.1 Method validation

Limits of detection (LOD), limits of quantification (LOQ) and linear dynamic ranges (LDR) were determined as described in Grasse et al. 2024. Depending on the linear dynamic range of each chemical, calibration solutions ranging from 0.05 to 100 ng/mL were prepared in ZFE matrix (8 ZFE per concentration) to determine the LOD and LOQ of each chemical. Each calibration standard had a water/MeOH ratio of 1:1 (v/v) and was measured in triplicates via HPLC-MS/MS. The LOD and LOQ values were calculated based on the peak areas using equations (1) and (2).

$$LOD = \frac{s_y}{b} * t * \sqrt{\frac{1}{n} + \frac{1}{m} + \frac{\bar{x}^2}{\sum_{i=1}^{n}(x_i - \bar{x})^2}} \quad (1)$$

$$LOQ = k * \frac{s_y}{b} * t * \sqrt{\frac{1}{n} + \frac{1}{m} + \frac{(k * x_N - \bar{x})^2}{\sum_{i=1}^{n}(x_i - \bar{x})^2}} \quad (2)$$

Legend:

s_y = standard deviation

t = value of t-distribution

b = slope of calibration curve

n = number of calibration values

m = number of replicates

x_i= measured value

$\bar{x}$ = average

Table S8: LOD, LOQ and LDR data for each study chemicals. Data for NMBA, BuMA, NIA, NDA and MA were collated from Grasse *et al.* 2024. For NBA, NPA, NPMA and NHMA data were determined in the present study.

Chemical	LDR [ng/mL]	LOD [ng/mL]	LOQ [ng/mL]	R^2
Acrylamide	10 - 250	0.43	1.98	0.9876
N'N-methylenebisacrylamide	1 - 20	0.80	3.70	0.9955
N-butoxy-methyl-acrylamide	0.5 - 35	0.45	2.10	0.9975
NIA	0.5 - 35	0.25	1.20	0.9935
N'N-Diethylacrylamide	1.0 - 35	0.68	3.16	0.9979
Methacrylamide	1.0 - 35	0.26	1.12	0.9939
N-benzacrylamide	0.5 – 100	0.19	0.92	0.9923
N-phenylacrylamide	0.5 – 100	0.20	0.94	0.9994
N-phenyl-methacrylamide	0.5 – 75	0.14	0.67	0.9918
N-(hydroxyphenyl)methacrylamide	1.0 - 50	0.21	1.02	0.9976

3.1.2 Matrix effects and recovery

Matrix effects and recoveries were determined according to Grasse et al. (2024) using triplicates prepared in ZFE matrix (8 ZFEs/replicate) for three standard concentrations of 5 ng/mL and 10 ng/mL at a lifestage of 96 hpf. Matrix effects seem to have an extremely strong influence on the analytical response of AA yielding in 1356 % matrix recovery and 731 % method recovery. For the remaining analytes, method recoveries ranged between 97 % and 140 % and matrix recoveries between 115 and 160 %. However, matrix effects were compensated using method-matched-calibration with representative ZFE extracts.

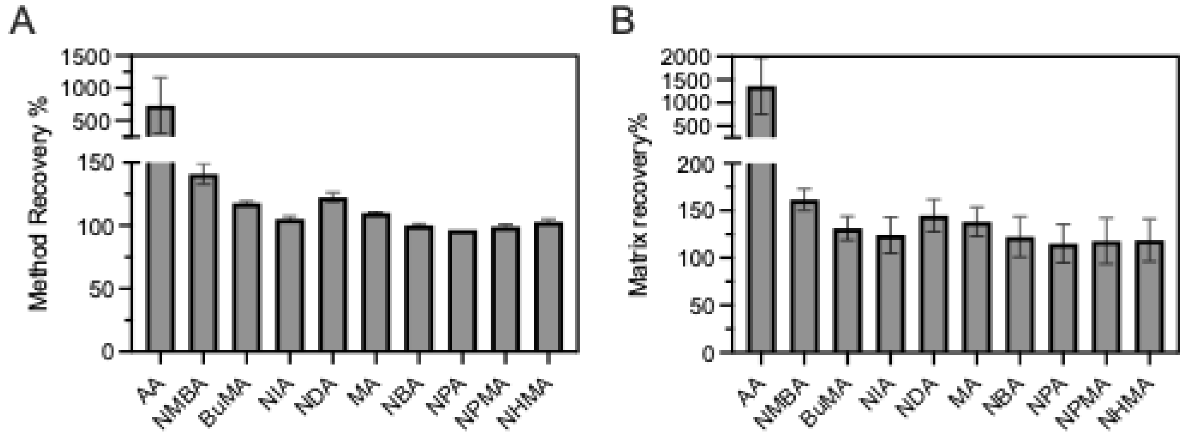

Figure S1: A Method recoveries and **B** Matrix recoveries of acrylamide (AA), *N'N'*-methylene-bisacrylamide (NMBA), *N*-butoxymethyl-acryalamide (BuMA), *N*-isobutoxymethyl-acrylamide (NIA), *N'N*-diethylacrylamide, methacrylamide (MA), *N*-benzylacrylamide (NBA), *N*-phenylacrylamide (NPA), *N*-phenyl-methacrylamide (NPMA) and *N*-(hydroxyphenyl)methacrylamide (NHMA) in ZFE matrix consisting of 8 ZFEs per replicate at a lifestage of 96 hpf.

3.2 UPLC-QTOF-MS

3.2.1 Detection of transformation products (TPs) using UPLC-QTOF-MS

For the detection and tentative identification of metabolites, ZFE samples were analyzed by ultra-performance liquid chromatography time-of-flight mass-spectrometry (UPLC-TOF-MS) using a AQUITY UPLC I-Class system (Waters) equipped with a HSS T3 column (100 x 2.1 mm, 1.7 µm) coupled to a XEVO G2S (Waters). The detailed instrumental conditions and the corresponding method can be found in the Table S9 . Samples were injected without further dilution in water/MeOH (1:1, v/v).

Table S9: Instrumental parameters of UPLC-qTOF-MS method for transformation product analysis according to Grasse et al.[1,21].

Parameter	Value
Flow rate	0.45 µL/min
Column temperature	45 °C
Capillary voltage	0.7 kV (positive mode)<br>- 1.3 kV (negative mode)
Source temperature	140 °C
Desolvation temperature	550 °C
Sampling cone voltage	20 V
Source offset	50 V
Cone gas	nitrogen
Collision gas	argon
Desolvation gas flow	950 L/h
Scan time	0.15 s
Collision energy	4 eV (molecular ions); 15 - 35 eV (fragments)
Mobile phase	A: MilliQ water + 0.1 % formic acid<br>B: methanol + 0.1 % formic acid
Solvent gradient	0 min 2% B, 12.25 min 99% B, 15.00 min 99% B;<br>15.10 min 2% B, 17.00 min 2% B.

3.2.2 Identification of TPs via suspect- and non-target-screening

To detect a wide range of TPs, a non-target screening approach using MarkerLynx (Waters, version 4.1) as described in Grasse et al. (2024) was conducted. Predicted exact masses of potential metabolites were screened in the m/z data set of exposed ZFEs and compared to the data of the control group of unexposed ZFEs. UPLC-HRMS data were processed in a retention time window of 1 to 10 min and a mass range of m/z 50 to 1200. The peak picking was performed with a 0.1 min deviation in retention time and 0.01 Da deviation in the exact mass.

Chemical formulas were generated with a mass tolerance of 5 ppm and elemental composition of C (0-100), H (0-100), N (0-20), O (0-20), P (0-1), S (0-20) and Na (0-2). The results were exported to Microsoft Excel for further statistical analysis. Results were visualized using GraphPad Prism. Furthermore, MS/MS fragmentations were considered for structure elucidation based on possible biotransformation pathways that may occur to the parental compounds.

Table S10: Parameter of MarkerLynx method used for detection of transformation products (collated from Grasse et al. 2024).

**Property**	**Value**
Function	1
Analysis type	Peak Detection
Initial retention time	1.0
Final retention time	10.0
Low mass	50.0
High mass	1200.0
XIC window (Da)	0.01
Use relative retention time	No
Apply smooting	No
Noise elimination level	0.0
Deisotope data	Yes
Replicate % Minimum	0.0

4. Biotransformation Prodcuts of ten (meth)acrylamides in the ZFE

All taurine conjugates shared diagnostic fragments (m/z 269.0262 and 124.0070) and a characteristic loss of *N*-acetylcysteine (–145.0192 Da), supporting structural identity via HRMS (SI1, **Figure S2**, **Tables S11**, **S12**). As an example, the MS/MS spectrum of BuMA_426 is provided in **Figure S2** in SI1.

Table S11: Summary of (meth)acrylamides and Respective Biotransformation Products Formed in *Danio rerio*. Mean values for the retention time (RT) and the mean bioconcentration factor (BCF, n = 3) are provided for each parent compound. RT = retention time. Oligomers of BuMA, NIA and NBA are excluded from this list and provided separatly in Table S12.

Compound	ESI mode	Formula [M] / Exact Mass of $[M+H]^+$ / $[M-H]^-$ (Δppm)	RT [min]	Elemental Change	Description	logP[i)] / logD_{lipw} (pH 7.4)[ii)]	Confidence level
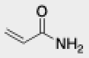 **AA** **BCF = N.A.**	N.A.	N.A.	N.A.	N.A.	parent compound	N.A.	N.A.
**AA_379**	positive	$C_{13}H_{23}N_4O_7S$ 379.1281 (-2.9)	1.23	$+C_{10}H_{17}N_3O_6S$	glutathione conjugation	-4.06	3
**AA_235**	positive	$C_8H_{15}N_2O_4S$ 235.0746 (-3.8)	2.15	$+C_5H_9NO_3S$	N-acetyl-cysteine conjugation	-1.76	3
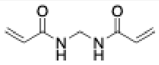 **NMBA** **BCF = 0.056 ± 0.032**	positive	$C_7H_{10}N_2O_2Na$ 177.0626 (-7.9)	2.94		parent compound	-0.56	1
**NBMA_791**	positive	$C_{27}H_{44}N_8O_{14}NaS_2$ 791.2305 (-1.4)	2.67	+2 $C_{10}H_{17}N_3O_6S$	2x glutathione conjugation	-7.6	3
**NMBA_481**	positive	$C_{17}H_{29}N_4O_8S_2$ 481.1418 (-3.0)	3.72+3.83	+ 2 $C_5H_9NO_3S$	2x N-acetyl-cysteine conjugation	-3.15	3
**NMBA_439**	positive	$C_{15}H_{27}N_4O_7S_2$ 439.1319 (-3.0)	2.61	$+C_5H_9NO_3S$; + $C_3H_7NO_2S$	N-acetyl-cysteine conjugation ; cysteine conjugation	-3.08	3

**NMBA_355**	positive	$C_{17}H_{28}N_5O_8S$ 462.165 (-1.3)	2.78	$+C_{10}H_{17}N_3O_6S$	glutathione conjugation	-3.83	3
**NMBA_340**	positive	$C_{12}H_{19}N_3O_5NaS$ 340.0937 (-1.8)	3.43; 3.53	$+C_5H_9NO_3S$	N-acetyl-cysteine conjugation	-1.6	3
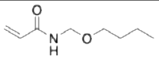 **BuMA** **BCF = 3.7 ± 1.7**	positive	$C_8H_{15}NO_2Na$ 180.0994 (-1.1)	6.31		parent compound	1.06	1
**BuMA_465**	positive	$C_{18}H_{33}N_4O_8S$ 465.2018 (-1.9)	5.08	$+C_{10}H_{17}N_3O_6S$	glutathione conjugation	-2.47	3
**BuMA_426**	negative	$C_{15}H_{28}N_3O_7S_2$ 426.137 (0.5)	5.43	$C_7H_{14}N_2O_5S_2$	N-acetyl-cysteine conjugation; taurine conjugation	-1.71	3
**BuMA_343**	positive	$C_{13}H_{24}N_2O_5NaS$ 343.1318 (-5.5)	6.18	$+C_5H_9NO_3S$	N-acetyl-cysteine conjugation	-0.23	3
**BuMA_279**	positive	$C_{11}H_{23}N_2O_4S$ 279.137 (-3.2)	4.58	$+ C_3H_7NO_2S$	cysteine conjugation	-0.16	3
**BuMA_373**	positive	$C_{14}H_{26}N_2O_6NaS$ 373.1401 (-4.0)	6.74	$+O + CH_3$ $+C_5H_9NO_3S$	aliphatic hydoxylation, N-methylation, N-acetyl-cysteine conjugation	-1.33	3
**BuMA_335**	negative	$C_{13}H_{23}N_2O_6S$ 335.128 (0.9)	4.00;4.37;5.12;5.75	$+O +C_5H_9NO_3S$	aliphatic hydoxylation, N-acetyl-cysteine conjugation	-1.56	3
**BuMA_384**	negative	$C_{13}H_{26}N_3O_6S_2$ 384.1254 (-2.3)	4.57	$+C_5H_{12}N_2O_4S_2$	cysteine conjugation; taurine conjugation	-1.64	3
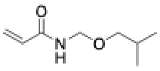 **NIA** **BCF = 0.39 ± 0.13**	positive	$C_8H_{15}NO_2Na$ 180.099 (-5.6)	6.27		parent compound	1.32	1
**NIA_319**	negative	$C_{13}H_{23}N_2O_5S$ 319.137 (6.3)	6.16	$+C_5H_9NO_3S$	N-acetyl-cysteine conjugation	-0.25	3
**NIA_426**	negative	$C_{15}H_{28}N_3O_7S_2$ 426.137 (0.2)	5.4	$+C_7H_{14}N_2O_5S_2$	N-acetyl-cysteine conjugation; taurine conjugation	-1.73	3
**NIA_384**	negative	$C_{13}H_{26}N_3O_6S_2$ 384.1285 (-1.3)	4.53	$+C_5H_{12}N_2O_4S_2$	cysteine conjugation; taurine conjugation	-1.66	4
**NIA_349**	negative	$C_{14}H_{25}N_2O_6S$ 349.144 (2.3)	6.7529	$+O + CH_3$ $+C_5H_9NO_3S$	aliphatic hydoxylation, N-methylation, N-acetyl-cysteine conjugation	-1.16	3

**NIA_277**	negative	$C_{11}H_{21}N_2O_4S$ 277.122 (-0.7)	4.53	+ $C_3H_7NO_2S$	cysteine conjugation	-0.18	3
**NIA_463**	negative	$C_{18}H_{31}N_4O_8S$ 463.1867 (0.9)	5.05	+$C_{10}H_{17}N_3O_6S$	glutathione conjugation	-2.48	3
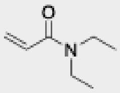<br>**NDA**<br>**BCF = 0.45 ± 0.03**	positive	$C_7H_{14}NO$ 128.1073 (-1.6)	5.64	N.A.	parent compound	0.3	1
**NDA_291**	positive	$C_{12}H_{23}N_2O_4S$ 291.138 (0 ppm)	5.74	+$C_5H_9NO_3S$	N-acetyl-cysteine conjugation	-0.68	3
**NDA_397**	negative	$C_{14}H_{25}N_2O_9S$ 397.128 (-0.3)	4.94	N.A.	N.A.	N.A.	4
**NDA_396**	negative	$C_{14}H_{26}N_3O_6S_2$ 396.125 (-3.0)	4.94	+$C_7H_{14}N_2O_5S_2$	N-acetyl-cysteine conjugation; taurine conjugation	-2.16	4
**NDA_433**	negative	$C_{17}H_{29}N_4O_7S$ 433.1768 (2.5)	4.65	+$C_{10}H_{17}N_3O_6S$	glutathione conjugation	-2.92	4
**NDA_249**	positive	C_{10}H21N2O3S 249.1262 (-4.4)	3.98	+ $C_3H_7NO_2S$	cysteine conjugation	-0.61	4
**NDA_100**	positive	$C_5H_{10}NO$ 100.0753 (-9.0)	2.95	- C_2H_4	hydrolysis	0.3	4
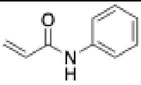<br>**NPA**<br>**BCF = 1.37 ± 0.30**	positive	$C_9H_{10}NO$ 148.0751 (-7.4)	6.19		parent compound	1.57	1
**NPA_416a**	positive	$C_{14}H_{16}N_3O_5Na_2S_2$ 416.0309 (-4.3)	4.44;6.12	+$C_5H_{12}N_2O_4S_2$	cysteine conjugation; taurine conjugation	-1.34	3
**NPA_347a**	positive	$C_{13}H_{21}N_2O_4Na_2S$ 347.1016 (-4.3)	6.95+6.84	+$C_5H_{12}N_2O_4S_2$ +O + CH_3	cysteine conjugation, aromatic hydroxylation, N-methylation	1.47	3
**NPA_416b**	negative	$C_{16}H_{22}N_3O_6S_2$ 416.0947 (-0.7)	5.38	+$C_7H_{14}N_2O_5S_2$	N-acetyl-cysteine conjugation; taurine conjugation	-1.41	3
**NPA_347b**	positive	$C_{13}H_{19}N_2O_5S_2$ 347.0722 (-3.7)	5.1619	+$C_4H_9NO_4S_2$			4
**NPA_325**	negative	$C_{14}H_{17}N_2O_5S$ 325.0858 (0.0)	5.03	+O +$C_5H_9NO_3S$	aromatic hydroxylation, N-acetyl-cysteine conjugation	-0.32	3

**NPA_311**	positive	$C_{14}H_{19}N_2O_4S$ 311.107 (1.3)	6.13	$+C_5H_9NO_3S$	N-acetyl-cysteine conjugation	0.07	3
**NPA_338**	negative	$C_{15}H_{16}NO_8$ 338.0868 (-2.4)	3.43	$+ C_6H_8O_7$	glucuronidation	-0.65	3
**NPA_242**	negative	$C_9H_8NO_5S$ 242.0121 (-0.8)	3.71	$+ O + SO_3$	sulfation	0.19	3
**NPA_455**	positive	$C_{19}H_{27}N_4O_7S$ 455.162 (1.8)	5	$+C_{10}H_{17}N_3O_6S$	glutathione conjugation	-2.16	3
**NPA_269**	positive	$C_{12}H_{17}N_2O_3S$ 269.0949 (-4.1)	4.44	$+ C_3H_7NO_2S$	cysteine conjugation	0.14	3
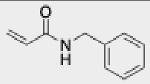 **NBA** **BCF = 0.94 ± 0.20**	positive	$C_{10}H_{12}NO$ 162.0909 (-6.2)	6.1		parent compound	1.76	1
**NBA_469**	positive	$C_{20}H_{29}N_4O_7S$ 469.1754 (-0.6)	5.02	$+C_{10}H_{17}N_3O_6S$	glutathione conjugation	-2.1	3
**NBA_341**	positive	$C_{15}H_{21}N_2O_5S$ 341.1162 (-2.6)	4.58;5.12;5.67	$+ O$ $+C_5H_9NO_3S$	aromatic hydroxylation, N-acetyl-cysteine conjugation	-0.25	3
**NBA_325**	positive	$C_{15}H_{21}N_2O_4S$ 325.1215 (-2.2)	6.07+6.14	$+C_5H_9NO_3S$	N-acetyl-cysteine conjugation	0.14	3
**NBA_283**	positive	$C_{13}H_{19}N_2O_3S$ 283.1103 (-4.6)	4.51	$+ C_3H_7NO_2S$	cysteine conjugation	0.21	3
**NBA_430**	negative	$C_{17}H_{24}N_3O_6S_2$ 430.1104 (-0.7)	5.35	$+C_7H_{14}N_2O_5S_2$	N-acetyl-cysteine conjugation; taurine conjugation	-1.34	3
**NBA_388**	negative	$C_{15}H_{22}N_3O_5S_2$ 388.0977 (-3.6)	4.5	$+C_5H_{12}N_2O_4S_2$	cysteine conjugation; taurine conjugation	-1.27	3
**NBA_444**	positive	$C_{23}H_{26}NO_8$ 444.1649 (-1.4)	7.02+7.07	$+ C_6H_8O_7$	dimerization, glucuronidation	1.41	3
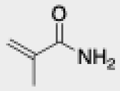 **MA** **BCF = 0.40 ± 0.52**	positive	C_4H_8NO 86.06	2.19		parent compound	0.29	1

**MA_249**	positive	$C_9H_{17}N_2O_4S$ 249.061 (-3.2)	2.85+2.96	$+C_5H_9NO_3S$	N-acetyl-cysteine conjugation	-1.26	3
**MA_354**	negative	$C_{11}H_{20}N_3O_6S_2$ 354.0786 (-2.3)	2.53	$+C_7H_{14}N_2O_5S_2$	N-acetyl-cysteine conjugation; taurine conjugation	-2.74	3
**MA_391**	negative	$C_{14}H_{23}N_4O_7S$ 391.1281 (-1.5)	2.17	$+C_{10}H_{17}N_3O_6S$	glutathione conjugation	-3.5	3
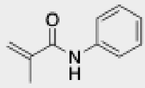 **NPMA** **BCF = 1.10 ± 0.40**	positive	$C_{10}H_{12}NO$ 162.092 (-2.5)	6.85		parent compounds	2.08	1
**NPMA_385**	positive	$C_{15}H_{19}N_2O_5Na_2S$ 385.0795 (0.3)	5.05;5.33;5.72;6.08	$+O +C_5H_9NO_3S$	aromatic hydroxylation (isomers), N-acetyl-cystein conjugation	0.25	3
**NPMA_299**	positive	$C_{13}H_{19}N_2O_4S$ 299.106 (-2.0)	4.66+4.73	$+ O + C_3H_7NO_2S$	aromatic hydroxylation, cystein conjugation	0.32	3
**NPMA_353**	negative	$C_{16}H_{18}NO_8$ 353.1 (-0.9)	4.19	$+ C_6H_8O_7$	glucuronidation	-0.3	3
**NPMA_256**	negative	$C_{10}H_{10}NO_5S$ 256.0283 (1.2)	4.4	$+ O + SO_3$	sulfation	0.18	3
**NPMA_323**	negative	$C_{15}H_{19}N_2O_4S$ 323.1074 (2.5)	6.61	$+C_5H_9NO_3S$	N-acetyl-cysteine conjugation	0.64	3
**NPMA_367**	negative	$C_{16}H_{19}N_2O_6S$ 367.0957 (-1.9)	6.22+6.52+6.81	$+ 2 O + CH_3 + C_5H_9NO_3S$	aromatic hydroxylation, N-methylation, N-acetyl-cystein conjugation	0.1	3
**NPMA_467**	negative	$C_{20}H_{27}N_4O_7S$ 467.1598 (-0.4)	5.48	$+C_{10}H_{17}N_3O_6S$	glutathione conjugation	-1.6	3
**NPMA_281**	negative	$C_{13}H_{17}N_2O_3S$ 281.096 (0.0)	5.16	$+ C_3H_7NO_2S$	cysteine conjugation	0.71	3
**NPMA_430**	negative	$C_{17}H_{24}N_3O_6S_2$ 430.1107 (0.0)	5.85	$+C_7H_{14}N_2O_5S_2$	N-acetyl-cysteine conjugation; taurine conjugation	-0.84	3
**NPMA_286**	negative	$C_{11}H_{12}NO_6S$ 286.0386 (0.3)	4.74	$+ 2O + SO_3 + CH_3$	aromatic hydroxylation, N-methylation, N-sulfation	-0.49	3
**NPMA_406**	positive	$C_{15}H_{24}N_3$O6S2 406.1107 (0.0)	4.78	$+ O +C_5H_{12}N_2O_4S_2$	aromatic hydroxylation, cysteine conjugation; taurine conjugation	-1.16	3
**NPMA_325**	negative	$C_{14}H_{17}N_2O_5S$ 325.0854 (-1.2)	5.04	$+ 2O + CH_3 + C_3H_7NO_2S$	aromatic hydroxylation, cysteine conjugation; N-methylation	0.24	3

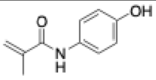 **NHMA** **BCF = 0.89 ± 0.20**	negative	$C_{10}H_{10}NO_2$ 176.0712 (0.0)	5.15		parent compound	2.18	1
**NHMA_367**	negative	$C_{16}H_{19}N_2O_6S$ 367.0961 (-1.9)	6.22+6.52+6.81	+ O + CH_3 + $C_5H_9NO_3S$	aromatic hydroxylation, N-methylation, N-acetyl-cystein conjugation	0.1	3
**NHMA_286**	negative	$C_{11}H_{12}NO_6S$ 286.0386 (0.3)	4.74	+ O + SO_3 + CH_3	aromatic hydroxylation, N-methylation, N-sulfation	-0.49	3
**NHMA_406**	positive	$C_{15}H_{24}N_3O_6S_2$ 406.1107 (0.0)	4.78;4.97	+$C_5H_{12}N_2O_4S_2$	aromatic hydroxylation, cysteine conjugation; taurine conjugation	-1.16	3
**NHMA_354**	positive	$C_{16}H_{20}NO_8$ 354.1183 (-0.6)	4.2	+ $C_6H_8O_7$	glucuronidation	-0.3	3
**NHMA_339**	negative	$C_{15}H_{19}N_2O_5S$ 339.1013 (-0.6)	5.04	+$C_5H_9NO_3S$	N-acetyl-cysteine conjugation	0.25	3
**NHMA_505**	positive	$C_{20}H_{29}N_2O_{11}S$ 505.1489 (-1.8)	3.3546	+ O + CH_3 + $C_6H_8O_7$ + $C_3H_7NO_2S$	aromatic hydroxylation, N-methylation, N-cystein conjugation	-1.72	3
**NHMA_325**	negative	$C_{14}H_{17}N_2O_5S$ 325.0854 (-1.2)	5.2	+ O + CH_3 + $C_3H_7NO_2S$	aromatic hydroxylation, cysteine conjugation; N-methylation	0.24	3
**NHMA_272**	negative	$C_{10}H_{10}NO_6S$ 272.0229 (0.0)	4.11	+ O + SO3	aromatic hydroxylation, sulfation	-0.21	3
**NHMA_485**	positive	$C_{20}H_{29}N_4O_8S$ 485.1706 (2.1)	4.15	+$C_{10}H_{17}N_3O_6S$	glutathione conjugation	-1.99	3
**NHMA_446**	negative	C17H24N3O7S2 446.1041 (-3.4)	3.23;3.45;3.96;4.36	+$C_7H_{14}N_2O_5S_2$	N-acetyl-cysteine conjugation; taurine conjugation	-1.23	3
**NHMA_299**	positive	C13H19N2O4S 299.1052 (-4.7)	3.49	+ $C_3H_7NO_2S$	cysteine conjugation	0.32	3

Table 12: UPLC-HRMS data and proposed identity of dimers and trimers of N-(butoxymethl)-acrylamide (BuMA), N-(isobutoxymethyl)-acrylamide (NIA) and N-benzyl-acrylamide (NBA) detected in ZFE extracts in positive and negative ionization modes.

**TP**	**Confidence level according to Schymanski et al.**	**Retention Time** [min]	**Ionization Mode**	**Molecular Ion (Δ ppm)** m/z	**Molecular Formula**	**Fragment Ions (Δ ppm)** m/z	**Proposed Structure**
BuMA (Parent)	1	6.31	positive	6.31 (-1.1)	$C_8H_{15}NO_2Na$	N.A.	N-(butoxymethyl)acrylamide
BuMA_266	4	10.5	positive	266.1726 (-3.0)	$C_{13}H_{25}NO_3Na$	N.A.	BuMA+OBu (Dimer)
BuMA_365	3	8.9	positive	365.2110 (0.0)	$C_{16}H_{33}N_2O_5S$	387.1947 (-2.1), 409.1750 (0.2), 342.1715 (-0.9), 326.1761 (-1.5), 302.0086 (3.6), 260.0391 (3.8), 214.0871 (1.4),188.0378 (-1.6), (-5.4), 165.991 (-4.9),142.032 (-6.7), 134.0267 (-7.8), 116.0161 (3.3), 88.0219 (-2.3)	BuMA+Cys+OBu
BuMA_481	3	9.95	positive	481.1953 (0.6)	$C_{19}H_{35}N_2O_7Na_2S$	296.183 (-1.7), 256.097 (-1.6), 168.0984 (-8.3)	BuMA + NAcCys + OBu + CH_2O
BuMA_467	3	7.7+7.9+8.1+9.0	positive	467.1799 (-0.9)	$C_{18}H_{33}N_2O_7Na_2S$	445.1975 (4.7), 371.1241 (-3.8), 338.1360 (-1.0), 316.1530 (-8.5), 282.1672 (2.5), 208.0090 (-5.3)	BuMA + O + NAcCys + OBu

BuMA_405	3	9.7	negative	405.207 (3.2)	$C_{18}H_{33}N_2O6S$	276.1638 (1.8), 230.1762 (2.6), 162.023 (3.1), 156.1028 (1.6)	BuMA + NAcCys + OBu
BuMA_10	3	9.2	positive	439.1848 (-0.9)	$C_{17}H_{33}N_2O_6Na_2S$	371.1968 (-3.2), 312.1576 (-2.9), 296.183 (-2.7), 188.037 (-5.8), 165.9904 (-6.6), 142.0316 (-7.7)	BuMA + Cys + OBu + O + CH_3
BuMA_409	3	8.9	positive	409.1740 (-0.2)	$C_{16}H_{31}N_2O_5Na_2S$	387.192 (-2.6), 342.1714 (-0.3), 326.1761 (-1.5), 217.0640 (-3.2), 214.0871 (1.4), 165.9910 (-6.0), 142.0320 (-4.9), 134.0267 (-6.7) , 116.016 (-8.6) , 88.0218 (-3.4)	BuMA + Cys + OBu
BuMA_470	4	8.7	negative	470.1992 (-0.6)	$C_{18}H_{36}N_3O_7S_2$	368.1268 (1.6), 227.0158 (2.0)	BuMA +Cys + Tau +OBu
NIA (Parent)	1	6.27	positive	180.0990 (-5.6)	$C_8H_{15}NO_2Na$		*N*-(isobutoxymethyl)-acrylamide
NIA_266	4	10.57	positive	266.1728 (-1.5)	$C_{13}H_{25}NO_3Na$	N.A.	NIA+OiBu
NIA_445	3	7.90+8.04+8.11+9.06	negative	445.1976 (-1.3)	$C_{18}H_{34}N_2O_7NaS$	282.1673 (-2.8), 240.0297 (-3.7)	NIA + NAcCys + OiBu
NIA_409	3	8.94	positive	409.1745 (-1.4)	$C_{16}H_{31}N_2O_5Na_2S$	387.1915 (-3.9), 342.1715 (0.0), 214.0866 (-0.9), 188.0371 (-5.3), 165.9904 (-6.6), 142.0316 (-7.7), 134.0264 (-9.0), 88.0214 (-8.0)	NIA + Cys + OiBu
NIA_470	3	8.73	Negative	470.1992 (-0.6)	$C_{18}H_{36}N_3O_7S_2$	312.0893 (-4.2), 227.0156 (-1.8), 209.9887 (-3.8), 194.0286 (5.2)	NIA+Cys+Tau+OiBu
NIA_461	4	9.22	Negative	461.1934 (-0.4)	$C_{18}H_{34}N_2O_8NaS$	N.A.	NIA + NAcCys + O + iOBu
NBA	1	6.10	positive	162.0909 (-6.2)	$C_{10}H_{12}NO$	108.0806 (-6.5), 84.0440 (-7.1)	N-benzyl-acrylamide

NBA_342	4	11.26+11.40	Positive	342.1852 (-1.8)	$C_{24}H_{24}NO$	N.A.	NBA + 2Benzyl (Trimer)
NBA_429	3	7.9109	Negative	429.1488 (-0.2)	$C_{22}H_{25}N_2O_5S$	322.0878 (-1.2), 300.1058 (0.3), 276.1000 (-0.4), 266.1181 (0.4), 254.1182 (0.4), 224.1072 (-1.3), 195.0806 (-2.1), 184.0038 (-3.3), 162.0223 (-1.2)	NBA+Benzyl+O+NAcCys
NBA_481	3	9.16+9.23	negative	481.1400 (-1.9)	$C_{23}H_{26}N_2O_6NaS$	327.1587 (-3.4), 354.1644 (-2.0), 284.1122 (4.6), 250.1230 (-0.8), 238.1232 (0.0), 162.0224 (-0.6)	NBA+O+Benzyl+NAcCys+O+CH3
NBA_444	3	7.02+7.07	positive	444.1649 (-1.4)	$C_{23}H_{26}NO_8$	268.1326 (-4.5), 290.1144 (-2.5), 197.0955 (-5.6)	NBA+Benzyl+O+Gluc
NBA_534	3	7.21+7.88+8.89+9.27+9.37	positive	534.2126 (-0.4)	$C_{30}H_{32}NO_8$	380.1616 (-2.6), 358.1797 (-2.9), 340.1686 (-4.4), 287.1419 (2.4), 193.101 (-9.3)	NBA+2 Benzyl + O + Gluc
NBA_553	3	7.57	negative	553.2006 (-0.4)	$C_{29}H_{33}N_2O_7S$	460.0645 (0.2), 424.1570 (-3.1), 406.1463 (-3.4), 372.1592 (-2.1), 354.1483 (-3.2)	NBA + 3O + NAcCys + 2 Benzyl
NBA_600	3	9.27+9.37+9.43	negative	600.1838 (-2.9)	$C_{31}H_{31}NO_{10}Na$	554.1775 (0.8), 532.1976 (-2.9), 378.1459 (-1.4), 356.165 (-4.7), 300.1374 (-2.9), 175.0238 (-7.7), 117.0179 (-5.2), 115.0025 (-6.1), 99.0076 (-5.4), 93.0335 (-5.9), 85.029 (4.9)	NBA + 2 Benzyl + 4O + Gluc + CH_3

NBA_520	3	8.61+8.72	negative	520.1574 (-0.4)	$C_{24}H_{30}N_3O_6S_2$	479.1306 (-1.0), 124.0067 (-0.8)	NBA + Cys + Tau + Benzyl

262

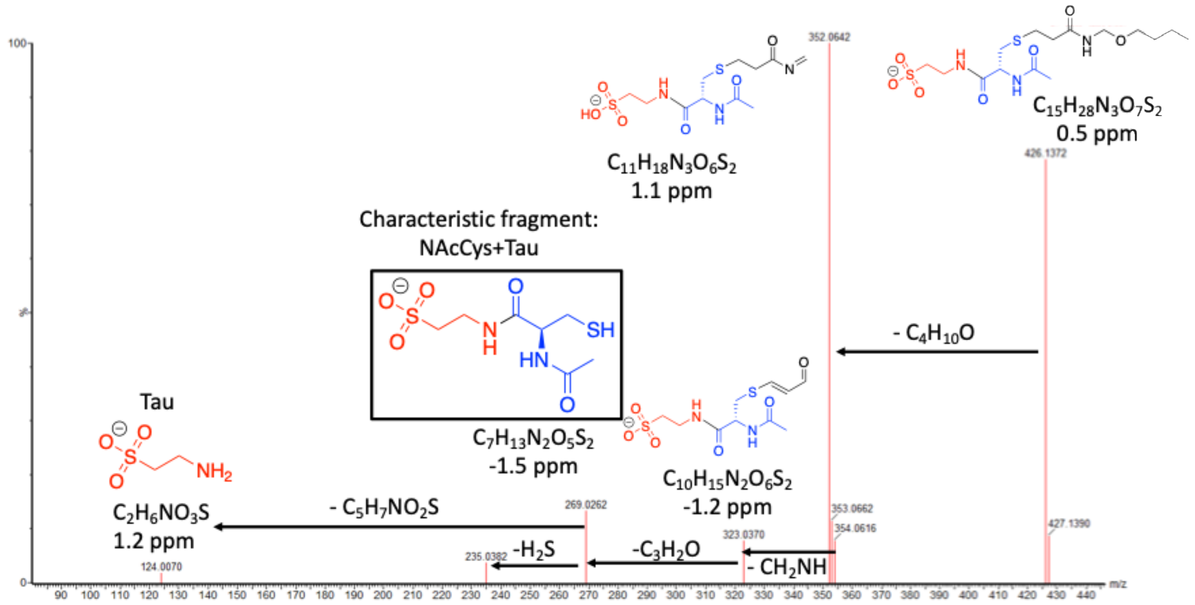

Figure S2: MS/MS fragment spectrum of BuMA conjugated to *N*-acetylcysteine (NAcCys) and taurine (Tau) (BuMA_426) with proposed chemical structures of fragments. The retention time was 5.43 min and data were recorded in negative ionization mode via UPLC-QTOF-MS.

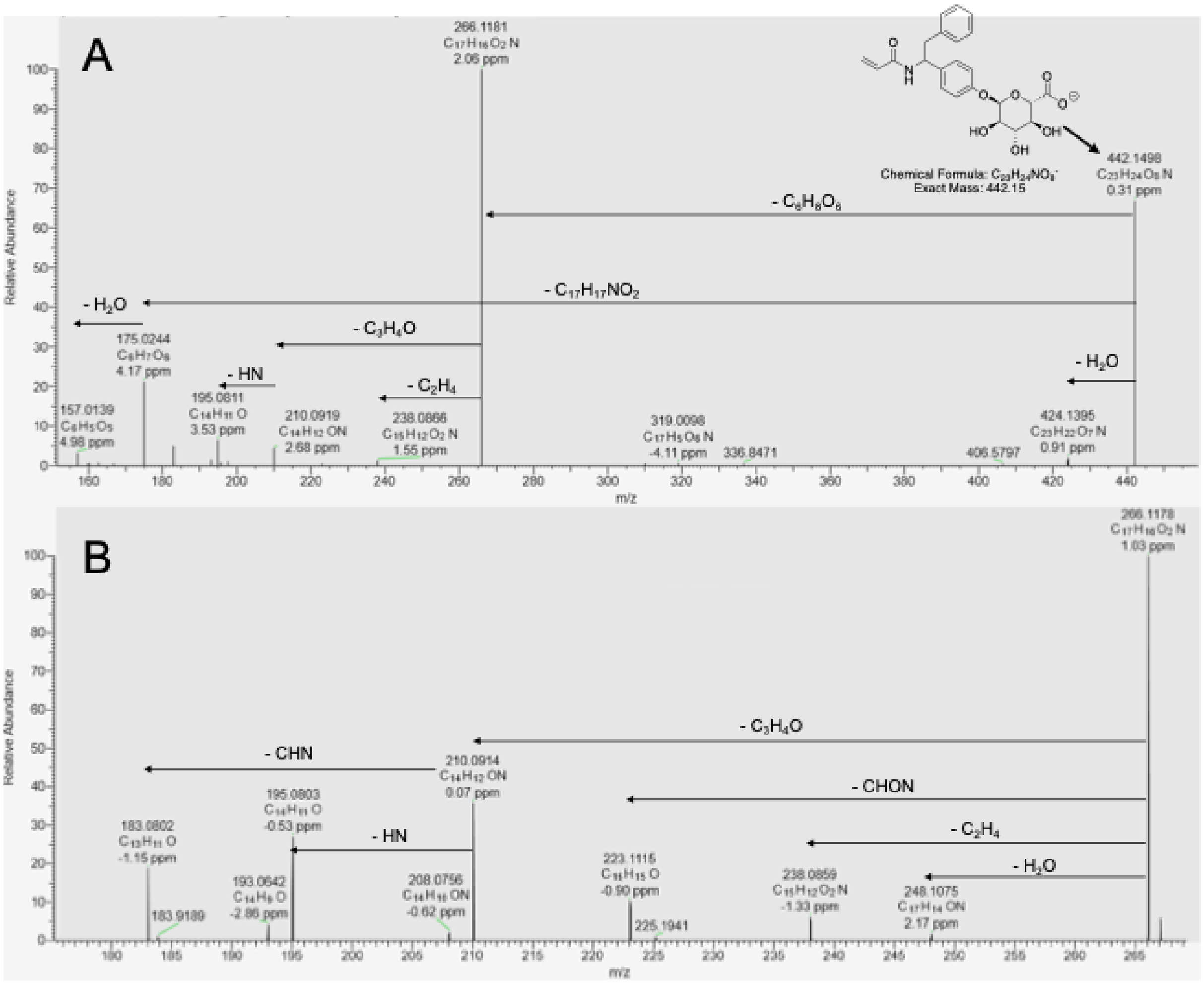

Figure S3: Fragment spectra of the glucuronated metabolite of NBA modified with one additional benzyl group (NBA_442). **A** MS/MS spectrum of NBA_442 (m/z 442.1498, $C_{23}H_{24}O_8N$, 0.31 ppm; retention time: 6.48 min). **B** MS^3 spectrum of the fragment m/z 266.1178 ($C_{17}H_{16}O_2N$, 1.03 ppm). High-resolution mass spectrometric data were generated using an Orbitrap IQ-X mass spectrometer (Thermo Fisher Scientific) in negative ionization mode coupled to reversed-phase liquid chromatography. Proposed chemical structure was assigned to the molecular ion.

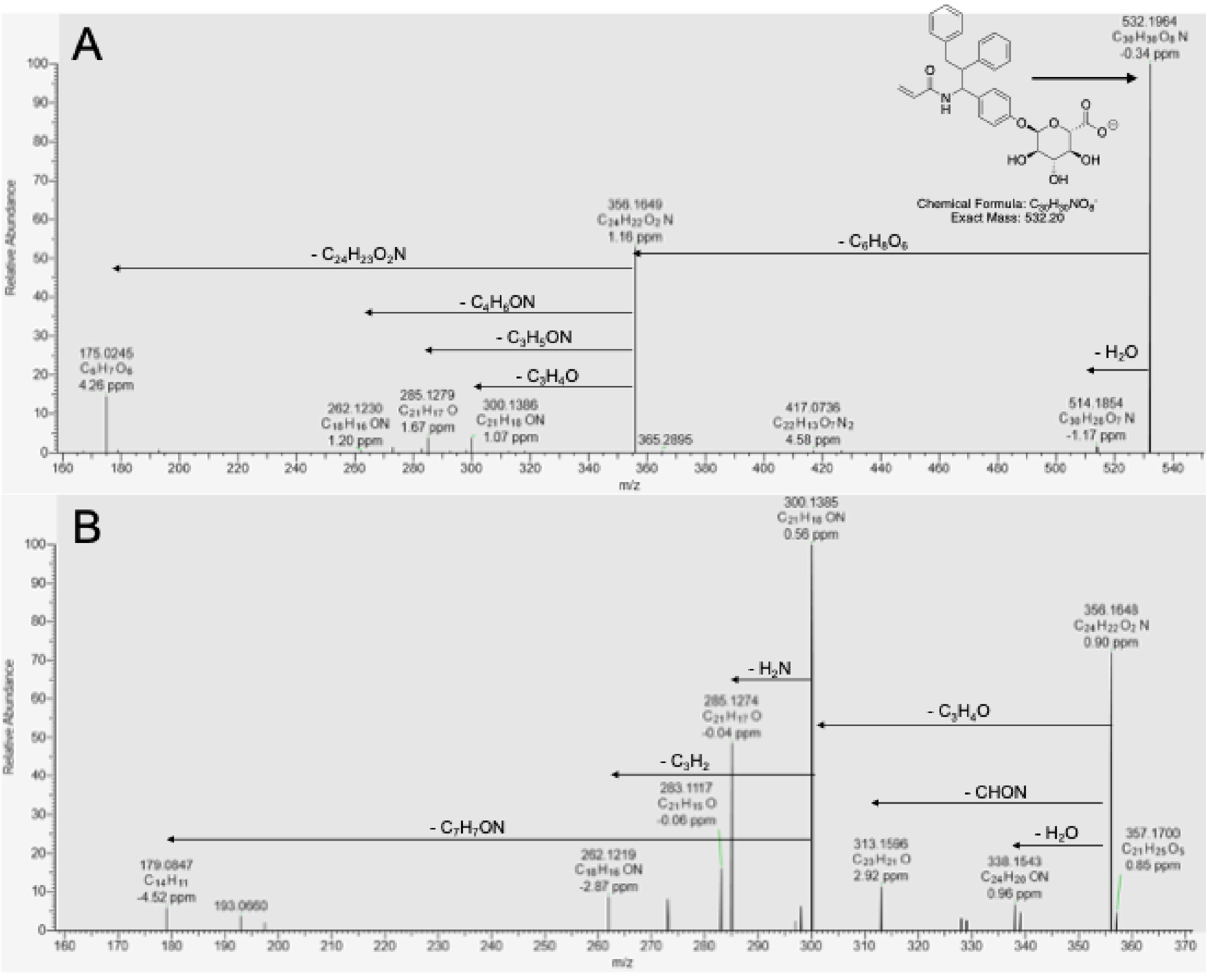

Figure S4: Fragment spectra of the glucuronated NBA metabolite (NBA_12) with two additional benzyl. **A** MS/MS spectrum of NBA_11 (m/z 532.1964, $C_{30}H_{30}O_8N$, - 0.34 ppm; retention time: 8.7 min). **B** MS[3] spectrum of the fragment m/z 356.1648 ($C_{24}H_{22}O_2N$, 0.90 ppm). High-resolution mass spectrometric data were generated using an Orbitrap IQ-X mass spectrometer in negative ionization mode coupled to reversed-phase liquid chromatography. Proposed chemical structure was assigned to the molecular ion.

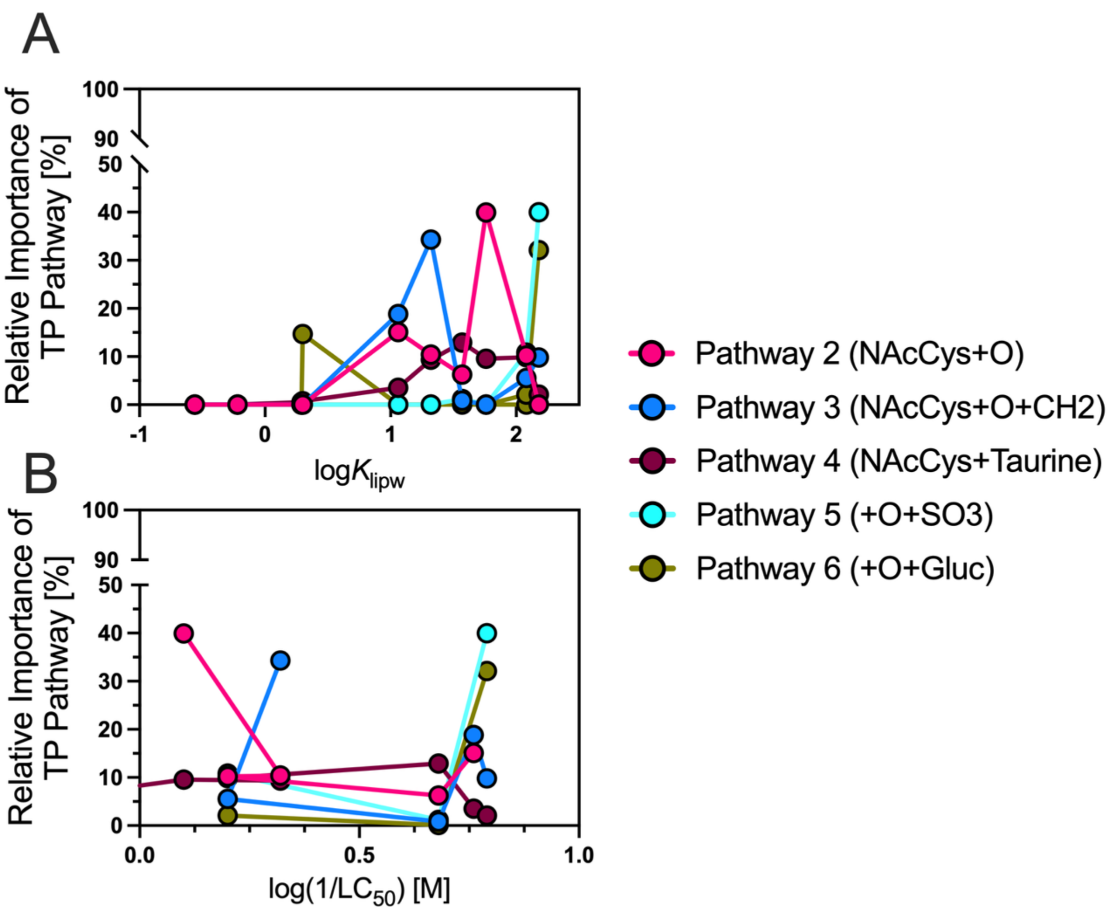

Figure S5: *Relationship between normalized peak areas of TPs of transformation pathways) with (A) Hydrophobicity (logK_{lipw}) and acute toxicity (LC_{50}).*
